# Development of P^N-Pd(II) Type Complexes Derived from Aliphatic Amines as Potential Antibacterial and Antifungal Agents

**DOI:** 10.3390/antibiotics15100968

**Published:** 2026-09-30

**Authors:** Michelle Acosta-Encinas, Andrés Amaya-Flórez, Jordi R. Galindo, Simón Hernández-Ortega, Maritza Lizeth Alvarez-Ainza, Josue Juarez, Adrián L. Orjuela, Jorge Alí-Torres, Alberto Aragón-Muriel, Viviana Reyes-Márquez, David Morales-Morales

**Affiliations:** 1 Departamento de Investigación en Polímeros y Materiales, Universidad de Sonora, Luis Encinas y Rosales S/N, Hermosillo 83000, Sonora, Mexico; a217204227@unison.mx; 2 Instituto de Química, Universidad Nacional Autónoma de México, Circuito Exterior S/N, Ciudad de México 04510, Coyoacán, Mexico; andresamaya93@comunidad.unam.mx (A.A.-F.); jordi.ruiz@cch.unam.mx (J.R.G.); simonho@unam.mx (S.H.-O.); 3 Departamento de Ciencias Químico-Biológicas, Universidad de Sonora, Luis Encinas y Rosales S/N, Hermosillo 8300, Sonora, Mexico; maritza.alvarez@unison.mx; 4 Departamento de Física, Universidad de Sonora, Luis Encinas y Rosales S/N, Hermosillo 83000, Sonora, Mexico; josue.juarez@unison.mx; 5 Plataforma de Biología y Química Computacionales, Instituto de Investigaciones Científicas y Servicios de Alta Tecnología (INDICASAT AIP), Panamá City 0843-01103, Panama; aorjuela@indicasat.org.pa; 6 Departamento de Química, Universidad Nacional de Colombia-Sede Bogotá, Bogotá 111321, Colombia; jialit@unal.edu.co; 7 Grupo de Investigación Bioquímicas (GIB), Departamento de Química, Universidad del Magdalena, Santa Marta 470004, Colombia; aaragonm@unimagdalena.edu.co

**Keywords:** antibacterial, antifungal, metallodrugs, iminophosphine, palladium(II) complexes

## Abstract

**Background/Objectives:** The development of new metallodrugs has represented an important milestone in recent years for their potential application in different therapeutic approaches. Therefore, the objective of this study was to synthesize a series of P^N iminophosphine Pd(II) complexes (**1Pd–3Pd**) incorporating different aliphatic amines. **Methods:** All complexes were characterized using several spectroscopic and analytical techniques, including ^1^H, ^13^C{^1^H}, and ^31^P{^1^H} NMR spectroscopy, mass spectrometry, FT-IR spectroscopy, and elemental analysis. In addition, complex **3Pd** was unambiguously characterized by single-crystal X-ray diffraction. **Results:** Supramolecular studies allowed the main noncovalent interactions responsible for stabilizing the crystal lattice of complex **3Pd** to be identified. These interactions were analyzed using Hirshfeld surface analysis, which revealed that C-H···π and C-H···Cl interactions contributed most significantly to the stabilization of the crystal lattice. All complexes exhibited stability in different solvents and positive lipophilicity values, with complex **3Pd** displaying the highest hydrophobicity. Through in vitro studies, the minimum inhibitory concentrations (MICs) and minimum fungicidal concentrations (MFCs) of all complexes were determined against different Gram-positive and Gram-negative bacterial strains, as well as fungal strains of Candida species. Complex **3Pd** exhibited the highest activity, particularly against *L. monocytogenes* (1.17 µg/mL), *P. aeruginosa* (4.68 µg/mL), and all fungal strains (18.75 µg/mL), displaying both bactericidal and fungicidal activity. Furthermore, in silico studies enabled the identification of the potential modes of action of the complexes against different molecular targets present in the bacterial and fungal strains. **Conclusions:** These results demonstrate the efficacy of the three synthesized complexes against different virulent strains exhibiting resistance to broad-spectrum drugs. Furthermore, the presence of the metal center, together with the incorporation of increasingly longer aliphatic chains, appears to enhance the antibacterial and antifungal activity of the complexes.

## 1. Introduction

The development of new metallodrugs with biological activity continues to be an important area of research in bioinorganic and medicinal chemistry [1,2,3]. These types of compounds offer several advantages over purely organic molecules, owing to their extensive structural diversity, which allows them to adopt a variety of molecular geometries and enables more efficient interactions with diverse biological targets [4,5,6]. The rational design of such structures represents a fundamental pillar, encompassing both the selection of the metal center and the type of ligands capable of modulating electronic and steric effects, thereby providing a synergistic effect aimed at enhancing their biological properties [7,8,9,10,11,12,13].

Among the most versatile ligands in the design of metallodrugs are Schiff bases, which are characterized by the presence of an azomethine group (C=N) that can efficiently coordinate to different metal centers [14,15,16]. Schiff bases can be readily modified by varying either the carbonyl compound or the amine precursor, constituting an efficient strategy for obtaining ligands with tunable steric and electronic properties. Furthermore, donor heteroatoms can be incorporated into the ligand framework, promoting the formation of more stable complexes [17,18,19]. For example, mixed P^N donor ligands are particularly attractive because they combine the σ-donor and π-acceptor properties of a phosphine group with the σ-donor effect of the nitrogen atom, enabling the formation of chelate complexes that allow fine control over the electronic environment at the metal center [20,21,22,23].

**Figure 1:**
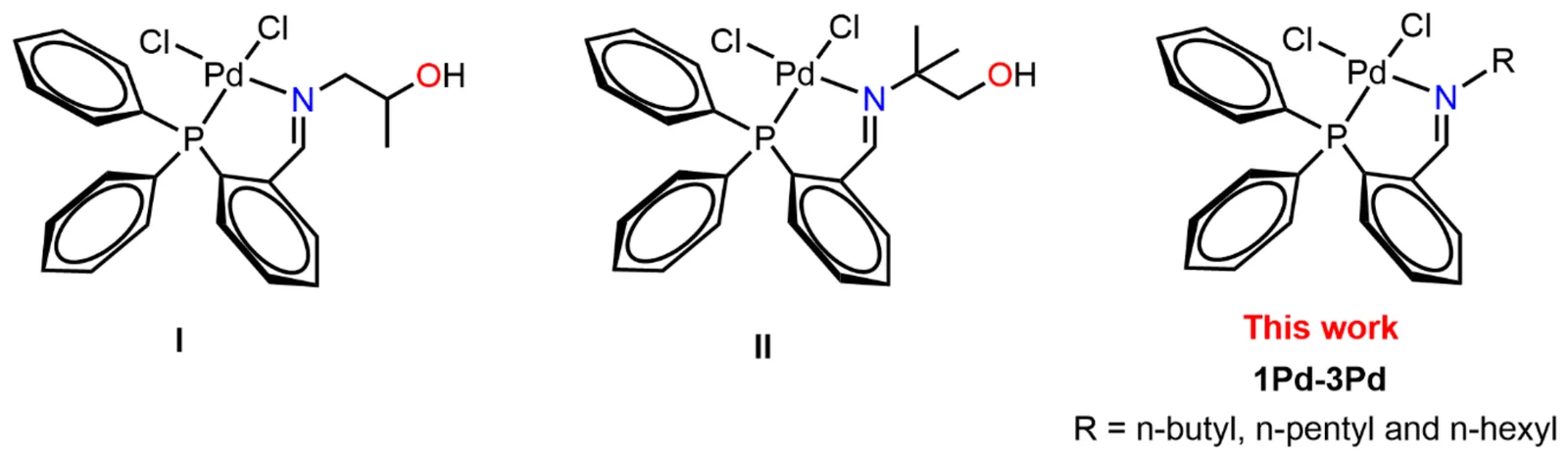
Molecular structures of P^N-Pd(II) complexes synthesized by our research group (**I** and **II**) with anticancer activity, and P^N-Pd(II) complexes evaluated in this study (**1Pd**–**3Pd**) as potential antibacterial and antifungal agents.

On the other hand, Pd(II) complexes have demonstrated promising biological properties due to their diverse mechanisms of action associated with interactions with different biological targets [24,25,26]. However, the higher reaction kinetics exhibited by these complexes often constitute a limitation, as they are commonly associated with toxic effects [27,28]. Nevertheless, the use of P^N-type ligands has proven to be an excellent strategy for modulating the kinetic properties of Pd(II) complexes. An example of this is provided by compounds recently reported by our research group, employing a series of amino alcohols, whose complexes **I** and **II** (Figure 1) showed IC_50_ values of 20.17 ± 0.6 µM (**I**) and 8.53 ± 1.9 µM (**II**) against the K562 cancer cell line [29].

Nowadays, the rapid emergence of bacterial resistance to conventional antimicrobial agents has created an urgent need to produce new compounds with alternative mechanisms of action. Antimicrobial resistance is currently recognized as one of the greatest global public health threats, as it increases mortality rates, prolongs hospital stays, and raises healthcare costs worldwide [9,30,31]. Therefore, the design of new metallodrugs capable of overcoming such resistance has emerged as a viable strategy, due to their ability to promote novel mechanisms of action that enable the inhibition of resistant bacterial strains [32,33,34,35].

Accordingly, with the aim of exploring their potential as antimicrobial agents, a series of P^N-Pd(II) complexes (**1Pd**–**3Pd**, Figure 1) were synthesized and evaluated against different Gram-positive and Gram-negative bacterial strains, as well as various fungal strains of the *Candida* species. This study was designed to investigate the possible synergistic effect between the palladium center and the structural variations in the P-N-type ligands, to correlate structural features with biological activity and identify promising candidates with enhanced antimicrobial properties. Furthermore, given the remarkable antibacterial and antifungal activities reported in the literature for Pd(II) complexes, molecular docking studies were conducted to investigate potential biological targets associated with these activities. Enzymes involved in ergosterol biosynthesis, cell-wall synthesis, DNA replication, and quorum-sensing pathways were selected as representative targets of the microorganisms evaluated experimentally.

## 2. Results

**Scheme 1:**
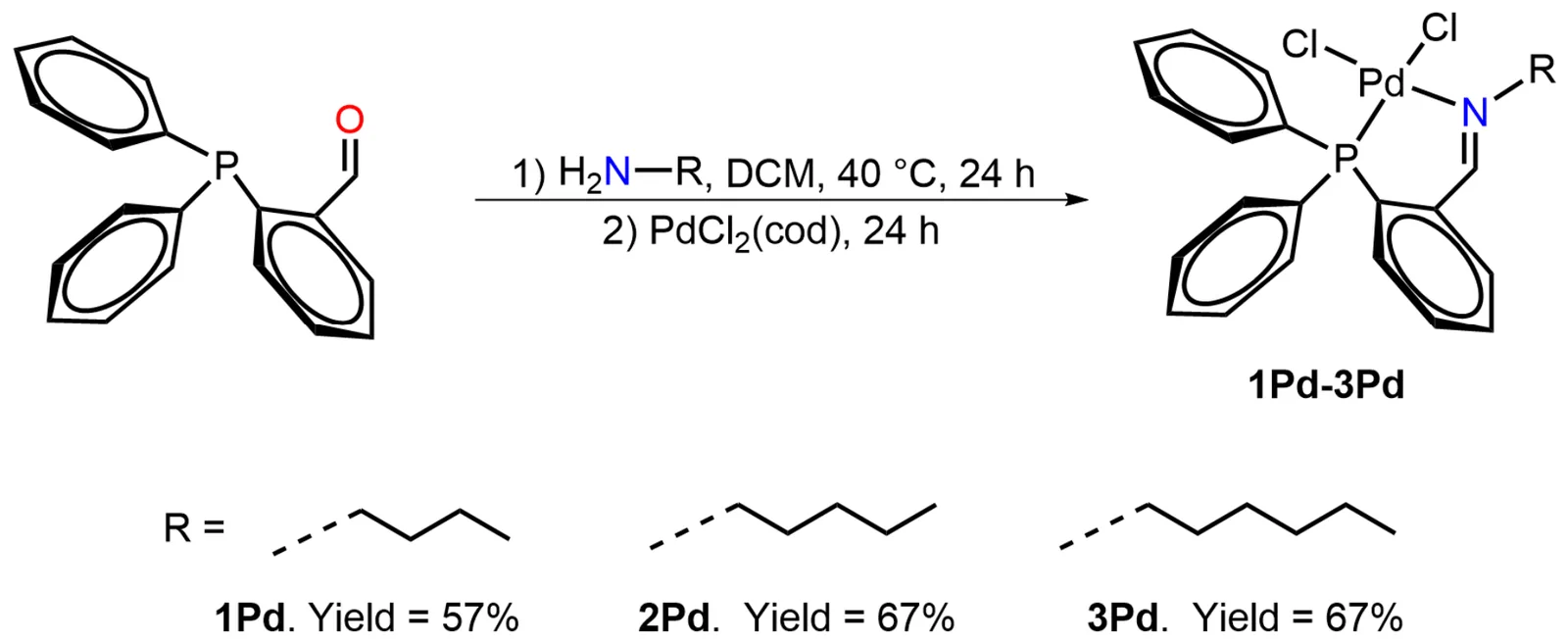
Synthesis of P^N-Pd(II) (**1Pd**–**3Pd**) derived from aliphatic amines.

The synthesis of complexes **1Pd**–**3Pd** was carried out in two stages following the methodology previously reported by our research group, with slight modifications [29]. For the preparation of the complexes (Scheme 1), the first step involved the formation of the imine ligands by dissolving 2-(diphenylphosphino)benzaldehyde and the corresponding aliphatic amines in dichloromethane (DCM). The reaction mixture was refluxed for 24 h, after which the metal precursor dichloro(1,5-cyclooctadiene)palladium(II), [PdCl_2_(cod)], was added in situ. The resulting solution was stirred at room temperature for an additional 24 h, affording yellow solids in moderate yields (57–67%). The main reason for not isolating the P^N ligands was their low stability upon exposure to air, as they were susceptible to oxidation. All complexes were characterized by NMR spectroscopy, FT-IR spectroscopy, mass spectrometry, elemental analysis, and single-crystal X-ray diffraction.

In the ^1^H NMR spectra of the three complexes, the signal corresponding to the iminic proton was observed in the range of 8.57–8.59 ppm, in agreement with values reported in the literature for this type of compound [20,29], along with the signals attributed to the methyl protons of the aliphatic chain. Regarding the ^13^C{^1^H} NMR spectra, the iminic carbon resonance for the three complexes appeared at 165 ppm. An increase in the number of signals in the aliphatic region (65.1–13.5 ppm) was observed, proportional to the length of the carbon chain. In the ^31^P{^1^H} NMR spectra, a single resonance was detected for the complexes in the range of 31.2–31.4 ppm, indicating that no significant electronic effects arise from increasing the length of the aliphatic chain (**S6**, **S9** and **S12**). Mass spectrometry analysis of the three complexes revealed the loss of a chloride fragment from the coordination sphere, with peaks corresponding to the molecular ions observed at *m*/*z* 486, 502, and 516 for **1Pd**, **2Pd**, and **3Pd**, respectively (Figures S10–S12). In the case of complexes **2Pd** and **3Pd**, m/z values of 1037 (**2Pd**, Figure S11) and 1065 (**3Pd**, Figure S12) were observed, which are consistent with their corresponding dimeric species. The formation of these species may be favored by the high temperatures employed during the acquisition of the mass spectra. FT-IR spectra show the characteristic bands associated with the imine bond (C=N) in the 1631–1633 cm^−1^ range (Figures S13–S15).

**Figure 2:**
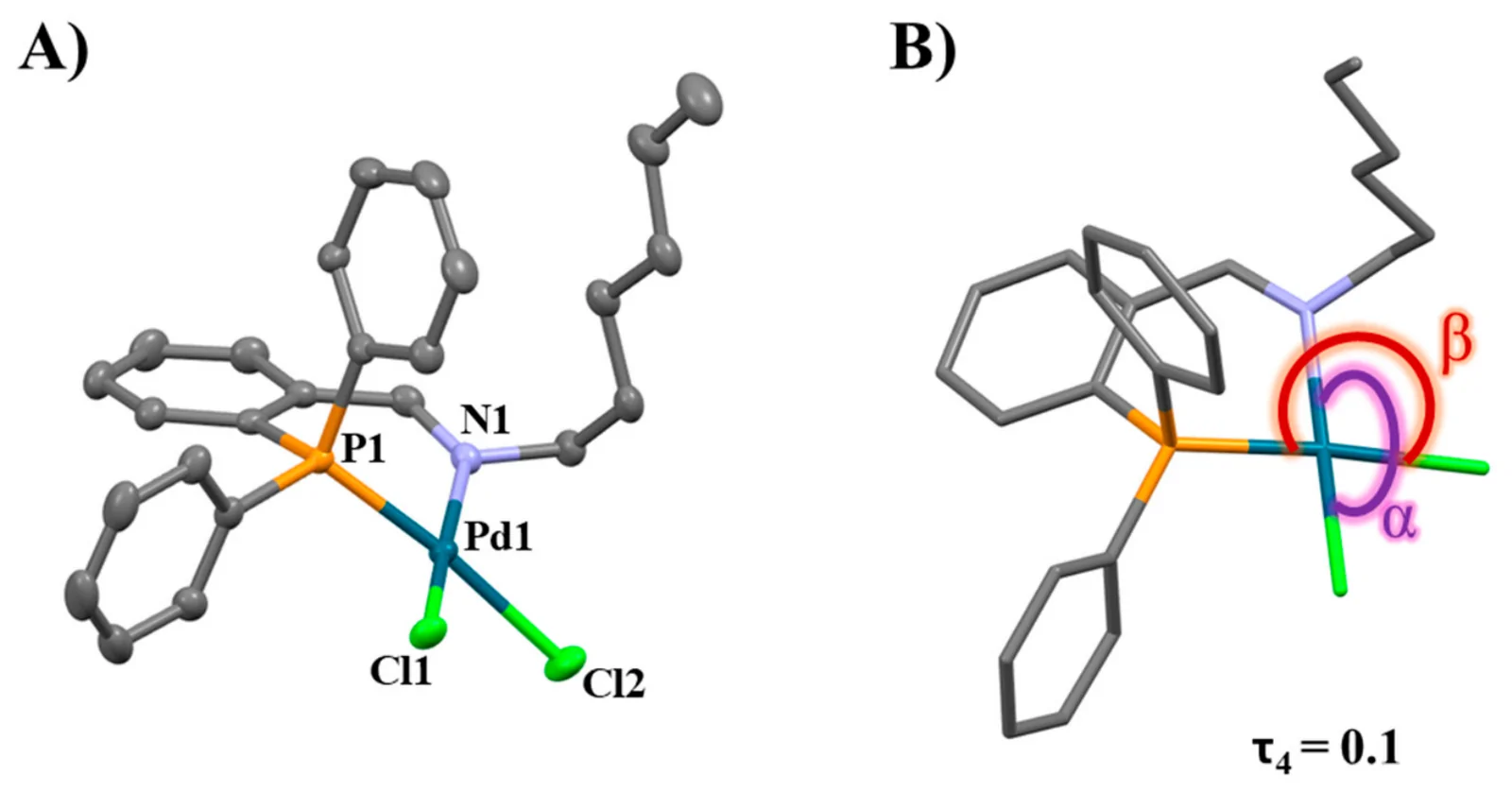
(**A**) Molecular structure of complex **3Pd** and (**B**) lateral view of complex **3Pd** showing the τ_4_ deviation from the square-planar geometry. Ellipsoids are drawn at the 50% probability level, and hydrogen atoms have been omitted for clarity. Select bond distances (Å) and angles (°) for **3Pd**: P(1)-Pd(1) 2.208(6), N(1)-Pd(1) 2.066(1), Cl(1)-Pd(1) 2.300(5), Cl(2)-Pd(1) 2.382(6), N(1)-Pd(1)-P(1) 88.8(4), N(1)-Pd(1)-Cl(2) 93.7(4), Cl(2)-Pd(1)-Cl(1) 90.6(2), P(1)-Pd(1)-Cl(1) 87.6(2), P(1)-Pd(1)-Cl(2) 174.3(2), N(1)-Pd(1)-Cl(1) 169.6(4).

The structure of complex **3Pd** was unequivocally determined by single-crystal X-ray diffraction (Table 1). Crystals were obtained from a saturated solution of DMSO and hexane (1:1) at room temperature. The structure crystallized in the monoclinic crystal system with space group P2_1_/n, and the molecular structure was solved and rendered using OLEX2 [36] v1.5 (Figure 2). The complex exhibits a square-planar geometry around the metal center. The iminophosphine ligand coordinates in a bidentate fashion through the nitrogen and phosphorus atoms, forming a six-membered chelate ring, while the chloride ligands adopt a *cis* arrangement, thereby completing the coordination sphere (Figure 2A). Intermolecular distances in the crystal structure were analyzed using Mercury [37] v2025.3.3.

The N-Pd bond distance is 2.066(1) Å, whereas the P-Pd bond distance is 2.208(6) Å, the latter being longer due to the larger atomic radius of the phosphorus atom, in agreement with previously reported related structures [20,29]. In addition, a *trans*-influence on the Pd-Cl bonds can be identified, as the Pd-Cl bond trans to the phosphine ligand is approximately 0.08 Å longer than the Pd-Cl bond *trans* to the imine ligand.

Furthermore, the bond angles around the Pd(II) center show a slight deviation from the ideal values of 90° and 180°, lying within the ranges of 88.8(4)–93.7(4)° and 169.6(4)–174.3(2)°, respectively. A deviation from the ideal square-planar geometry can be assessed using the Okuniewski parameter (τ_4_), which is calculated as the sum of the two largest angles (α and β) around the metal center (Figure 2B), subtracted from 360°, and divided by 141° [38,39]. A τ_4_ value of 0 corresponds to a perfect square-planar geometry, whereas τ_4_ = 1 indicates a tetrahedral geometry. The τ_4_ value calculated for **3Pd** is 0.1, reflecting its close approximation to a square-planar geometry.

### 2.1. Supramolecular Analysis

From a supramolecular analysis, the principal supramolecular interactions that stabilize the crystal lattice can be identified. Although this type of study is conducted in the solid state, it also provides insight into the potential interactions that the complex may establish with different biological targets, thereby offering and indicating the key interactions that this complex could potentially exhibit in solution with relevant biological targets. The interactions of complex **3Pd** are summarized in Table 2 and are described using graph-set descriptors.

**Figure 3:**
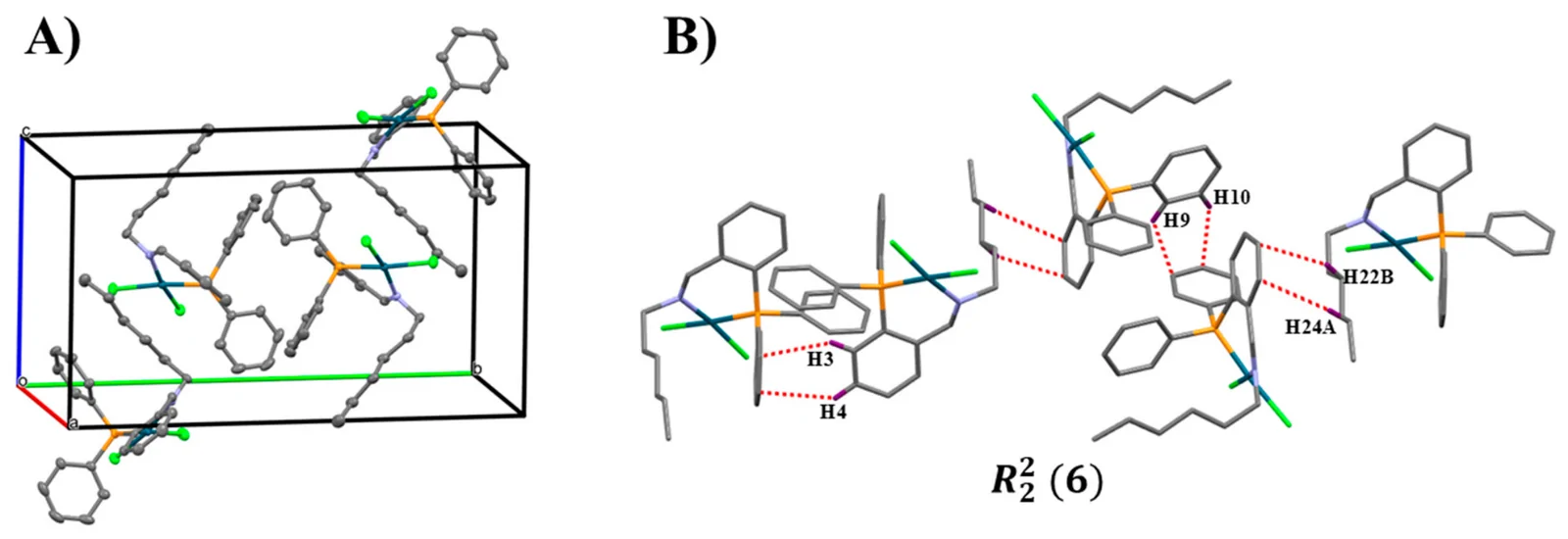
Non-covalent interactions present in (**A**) the crystal lattice of complex **3Pd** and (**B**) the $R_{2}^{2}\left(6\right)$ ring motif. Hydrogen atoms not involved in these interactions were omitted for clarity.

The unit cell consists of four molecules of complex **3Pd** (Figure 3A), whose crystal lattice is stabilized by different non-covalent CH···C interactions displaying an $R_{2}^{2}\left(6\right)$ ring motif, primarily generated by the aromatic rings of the phosphine moiety [1 − x, 1 − y, 1 + z] and the aliphatic chain [−1/2 + x, 1/2 − y, 1/2 + z] (Figure 3B).

**Figure 4:**
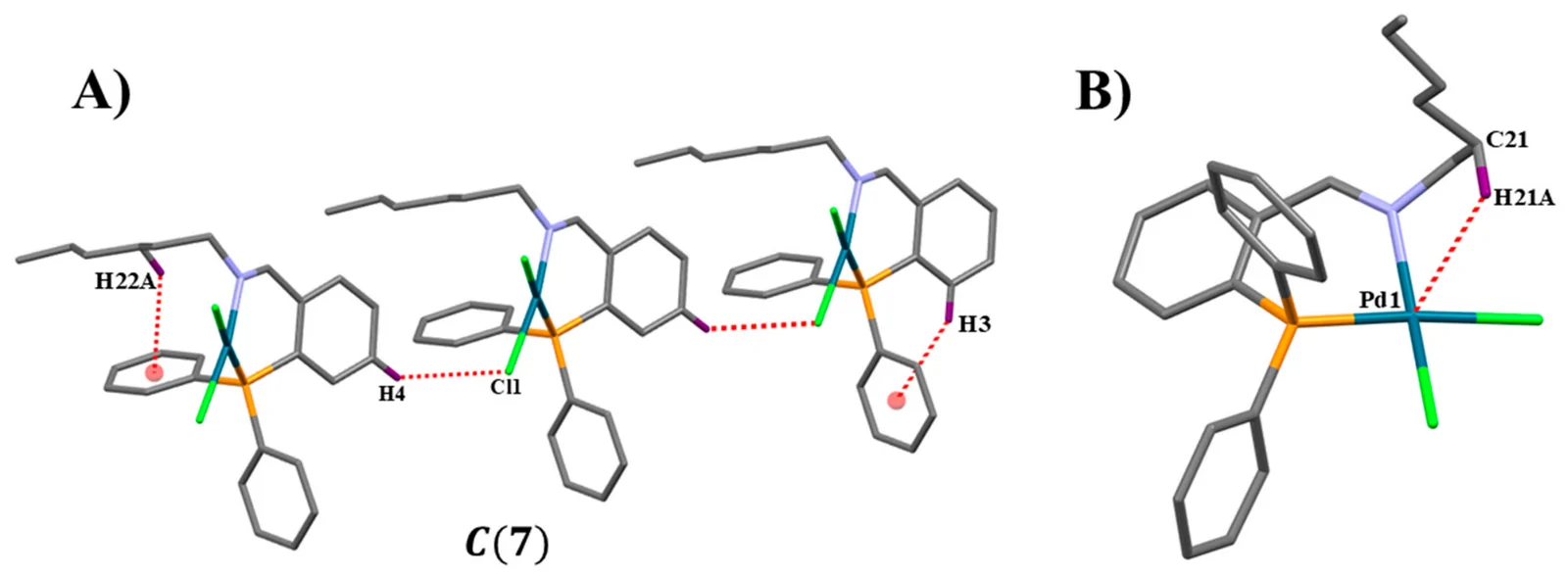
Non-covalent interactions of complex **3Pd**: (**A**) C(7) chain motif and intramolecular edge-to-face CH···π interactions and (**B**) anagostic interaction between C21-H21A···Pd. Selected hydrogen atoms were omitted for clarity.

Additionally, intramolecular edge-to-face CH···π interactions can be observed between the aromatic rings of the phosphine moiety and the H3 atoms [1 − x, 1 − y, 1 − z], as well as the aliphatic protons H22A [1 − x, 1 − y, 1 − z]. Similarly, polymeric CH···Cl interactions displaying a C(7) chain motif are identified (Figure 4A). Furthermore, the presence of axial H···Pd anagostic interactions relative to the coordination sphere can be inferred. This interaction occurs between the H21A proton of the α-carbon in the aliphatic chain and the palladium center, at 2.837 Å and with an angle of 114°, values that fall within the optimal range for this type of electrostatic interaction to occur (Figure 4B) [40,41,42].

### 2.2. Hirshfeld Surface

**Figure 5:**
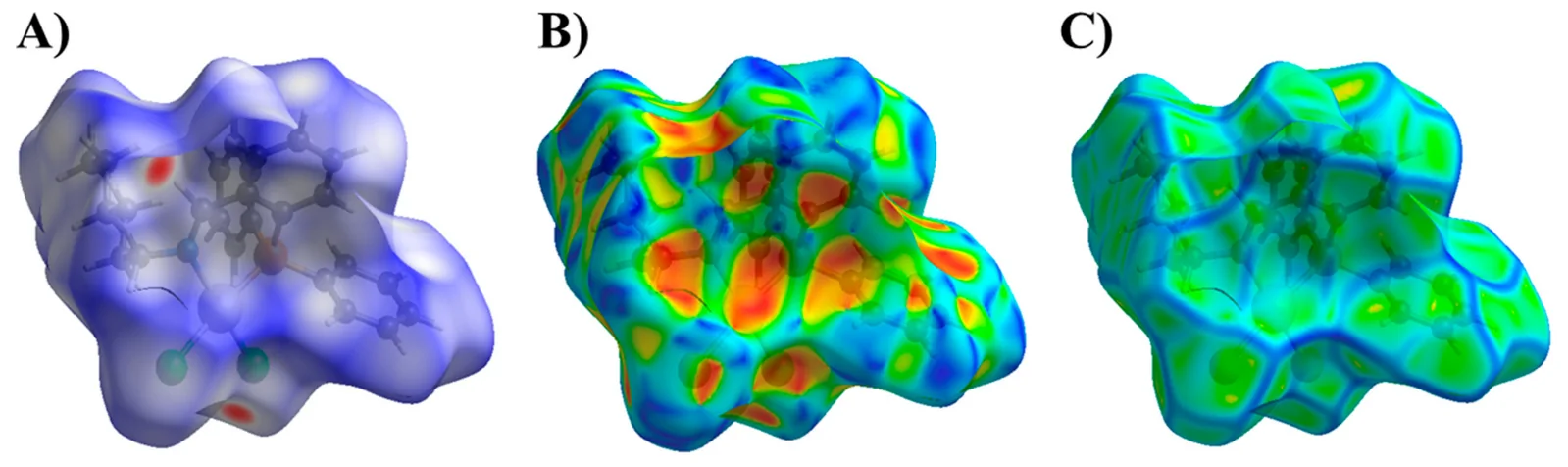
Supramolecular analysis based on Hirshfeld surfaces mapped over (**A**) d_norm_, (**B**) shape index, and (**C**) curvedness for complex **3Pd**.

**Figure 6:**
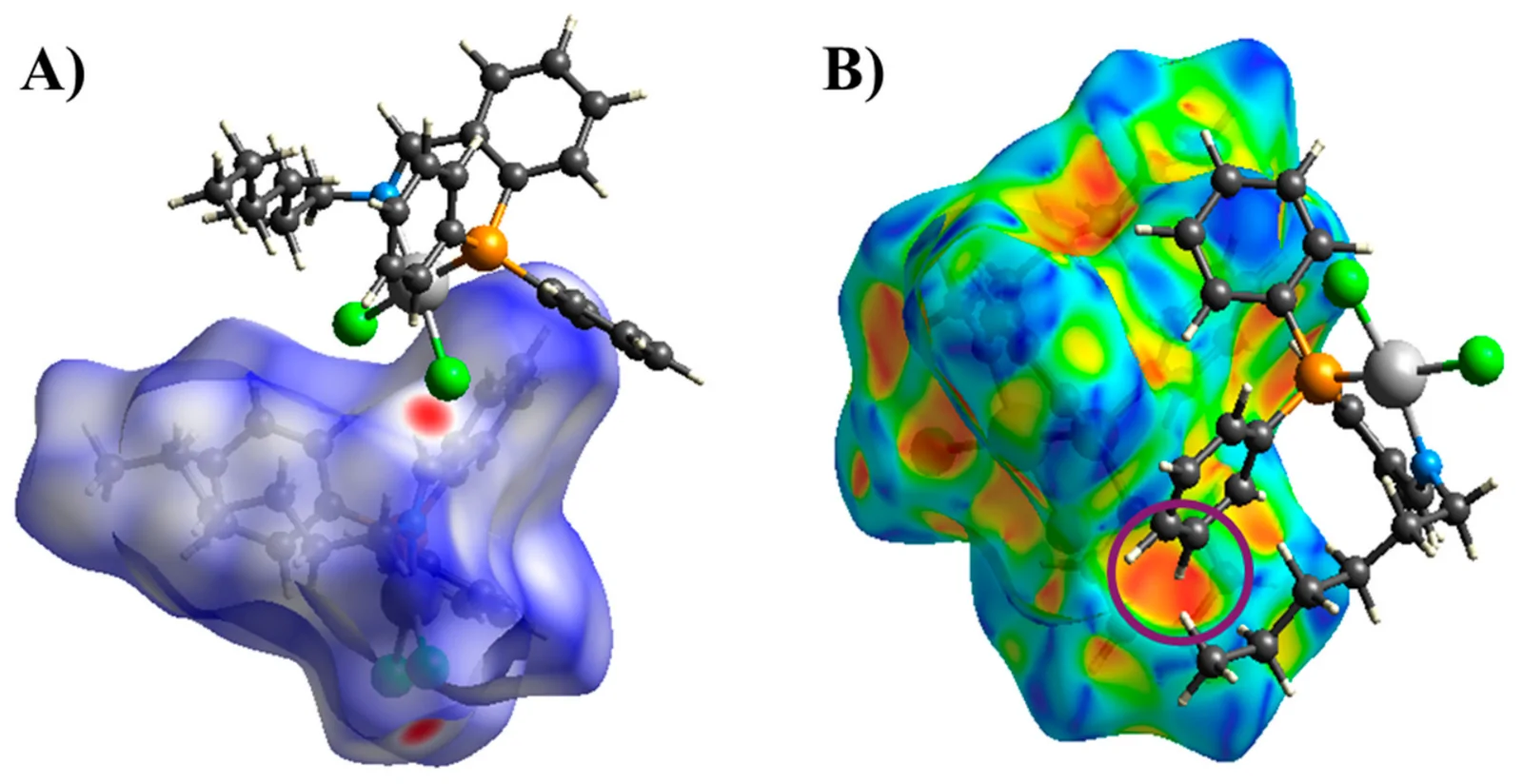
Hirshfeld surface mapped over (**A**) d_norm_ and (**B**) shape index for **3Pd**, revealing the most relevant interactions of the complex.

Hirshfeld surface analysis provides a deeper understanding of the supramolecular interactions within a crystal lattice, offering both quantitative and qualitative information and enabling visualization of close intermolecular contacts. These surfaces were generated using the Crystal Explorer software (version 21) [43], yielding three-dimensional surfaces mapped over d_norm_, shape index, and curvedness properties (Figure 5A–C). Two intense red regions are observed on the d_norm_ surface, corresponding to short intermolecular contacts that are shorter than the sum of the van der Waals radii (Figure 5A). These regions are located over hydrogen and chlorine atoms, suggesting the presence of CH···Cl hydrogen bonding interactions (Figure 6A). Moreover, the shape index allows identification of characteristic packing modes, such as planar stacking arrangements and the presence of adjacent molecules in close contact. This is evidenced by red regions distributed over the aromatic rings (Figure 5B), highlighting edge-to-face CH···π interactions (Figure 6B). The triangular patterns observed on the aromatic rings in the curvedness surface are indicative of π···π stacking interactions. Since such interactions are absent in the crystal lattice of complex **3Pd**, no broad and flat regions are observed on the curvedness surfaces (Figure 5C).

**Figure 7:**
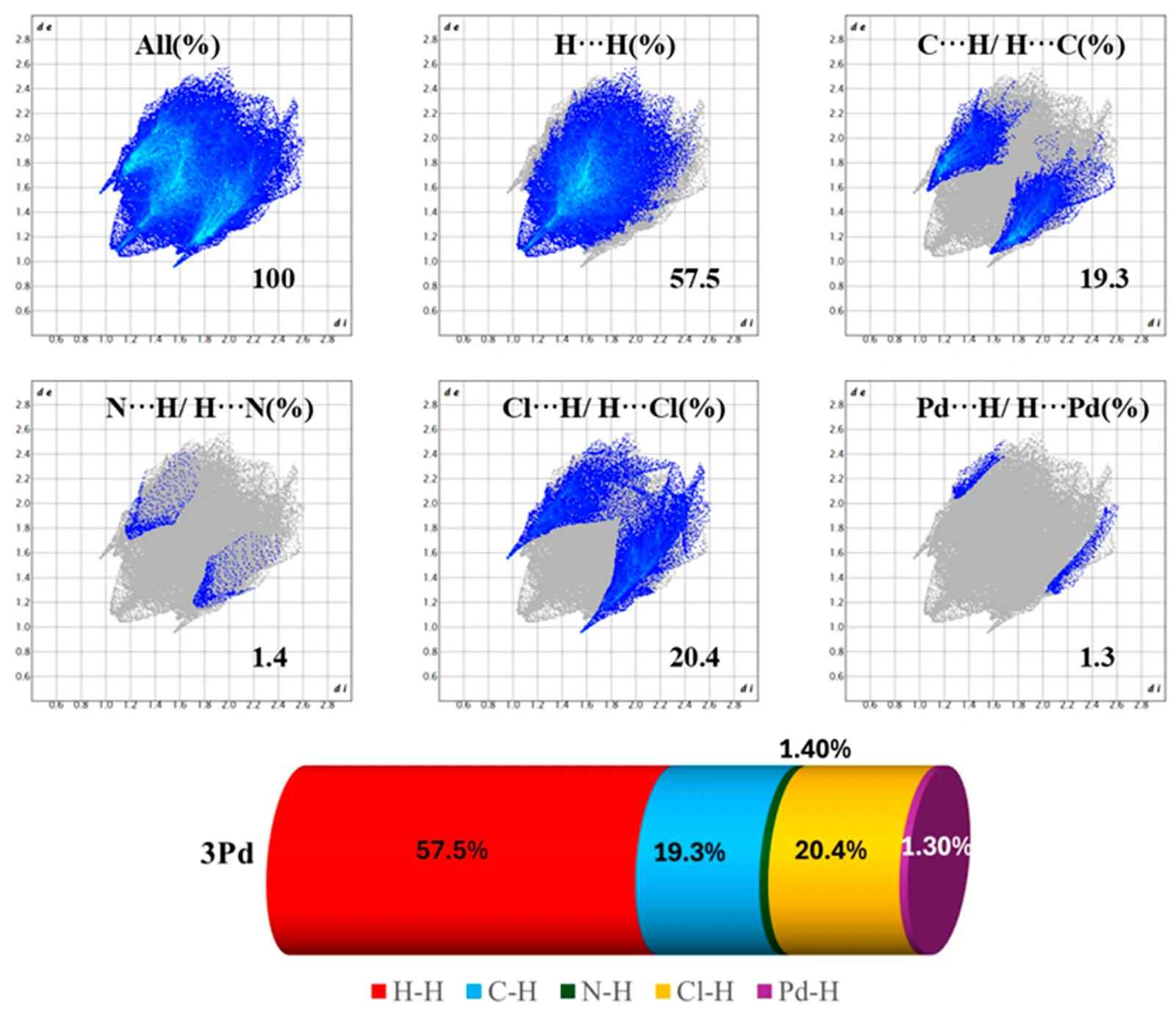
Representation of the fingerprint plot of non-covalent interactions in complex **3Pd** and a chart showing the percentages of the observed contacts within the complex.

The supramolecular analysis was complemented with two-dimensional (2D) fingerprint plots, which are generated by combining the distances from each point on the internal (d_i_) and external (d_e_) surfaces of the Hirshfeld surface. These plots provide information on the distribution of intermolecular contacts, highlighting the types of interactions that predominate within the crystal lattice. Through decomposed plots, individual atom–atom interactions can be categorized and quantified. For complex **3Pd**, the plots indicated that H···H interactions are the most prevalent, accounting for 57.5% of the intermolecular contacts. Additionally, Cl···H/H···Cl contacts contribute significantly to the total interactions (20.4%). Interactions such as N···H/H···N and C···H/H···C contribute the least to the total contacts within the crystal lattice (1.4% and 19.3%, respectively). Furthermore, Pd···H/H···Pd interactions, although minor (1.3%), are also present, which would further corroborate the presence of anagostic interactions, as previously observed (Figure 7).

The non-covalent interactions analyzed above provide important insight into the supramolecular behavior of complex **3Pd**, which may be relevant at the cellular level and facilitate interactions with various biological targets for potential inhibition.

### 2.3. Stability Studies

**Figure 8:**
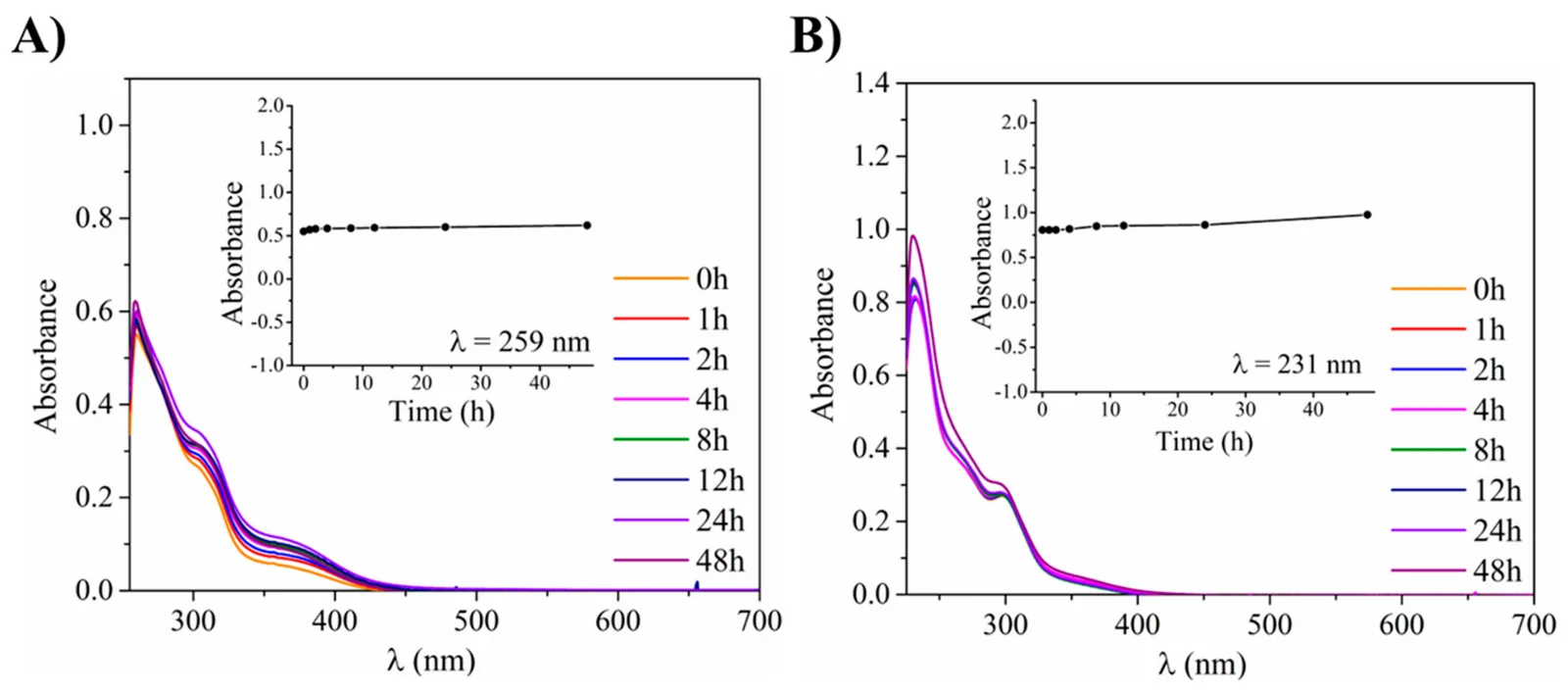
UV/Vis stability assays of complex **3Pd** at 25 µM, room temperature, over different time intervals in: (**A**) DMSO. Inset: Plot of the time variation in the absorption spectra at 259 nm. (**B**) Milli-Q water/DMSO (0.1% *v*/*v*). Inset: Plot of the time variation in the absorption spectra at 231 nm.

Drug stability plays an important role in cellular internalization and in the interaction with specific biological targets [44,45]. Therefore, stability studies were carried out for all complexes in two different solvents to evaluate the possible effects of the medium on the stability of each compound. Stability studies were carried out using UV–Vis spectroscopy, as this technique provides valuable information on changes in absorbance and their potential relationship to structural changes in the compounds. Although this technique does not provide unequivocal evidence of the specific structures formed over time, it offers valuable insights into the possible transformations occurring in the compounds under investigation. Solutions were prepared at 25 µM in DMSO and in Milli-Q water containing 0.1% (*v*/*v*) DMSO. Stability measurements were performed using UV-Vis spectroscopy at room temperature and different time intervals: 0, 1, 2, 4, 8, 12, 24, and 48 h. A hyperchromic effect was observed for all complexes as time increased. In the case of the **3Pd** complex, it remained stable in both solutions, showing degradation percentages of 11% in DMSO (Figure 8A) and 17% in Milli-Q water (Figure 8B).

Regarding the stability of complexes **1Pd** and **2Pd**, **1Pd** exhibited greater stability in aqueous medium, with a degradation percentage of 13%, compared to DMSO, where a degradation of 39% was observed. This behavior may be attributed to the higher coordinating ability of DMSO (Figure S16). Regarding **2Pd**, it was found to be stable in both media, exhibiting degradation percentages identical to those of **3Pd**, namely 11% in DMSO and 17% in aqueous medium (Figure S17). It is important to note that the changes in absorbance intensities observed in the different media could be attributed to coordination of DMSO or water molecules [46,47]. Since these solvents can act as coordinating ligands, they could potentially replace the chloride ions within the coordination sphere. On the other hand, the absence of new absorption bands over the course of the experiments provides evidence suggesting that the complexes did not undergo decomposition. Likewise, conducting these types of stability assays on Pd(II) complexes containing imine moieties is important, as these compounds may undergo decomposition under thermal, photochemical, or environmental conditions. The results of the present study indicate that complexes **1Pd**–**3Pd** exhibited sufficient stability to be considered suitable for use in biological assays.

### 2.4. Lipophilicity Assay

**Figure 9:**
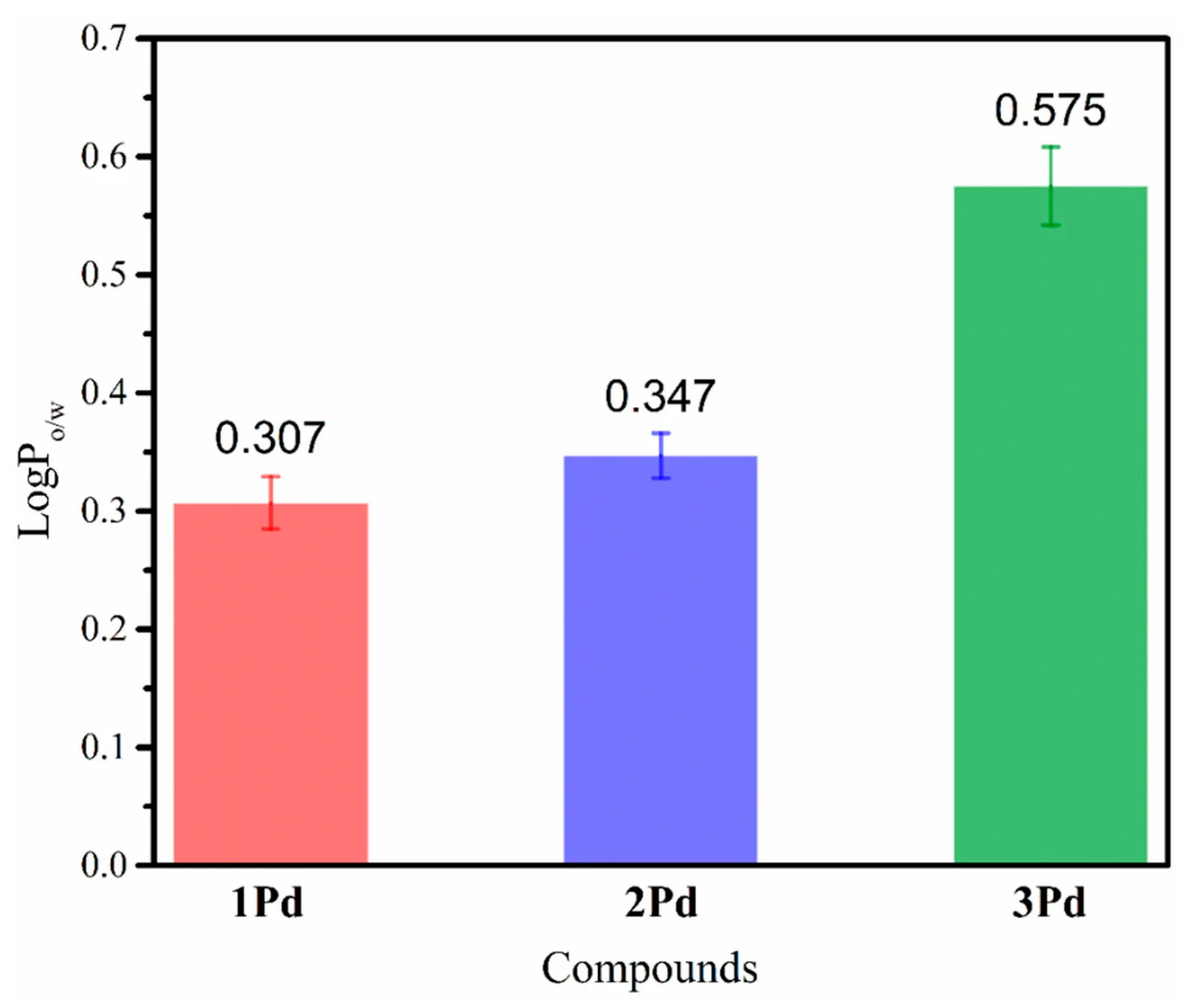
LogP_o/w_ values of the P^N-Pd(II) complexes **1Pd**–**3Pd**.

The lipophilicity of metal-based compounds plays an important role in cellular uptake, as well as in their cytotoxic and antimicrobial properties [48,49]. For metal complexes, an increase in lipophilicity generally enhances these biological properties. This parameter is used to determine the affinity of compounds for hydrophilic or lipophilic environments; negative values indicate hydrophilicity, whereas positive values denote a preference for the lipophilic phase, and values close to zero suggest a balanced affinity for both phases [50]. Therefore, lipophilicity studies were carried out and expressed as the octanol/water partition coefficient (LogP_o/w_), which relates to the ability of a given compound to permeate cellular membranes. The “shake-flask” method was employed using UV-Vis spectroscopy for the determination of the partition coefficient. As shown in Figure 9, all complexes exhibited positive LogP_o/w_ values, indicating their lipophilic nature. Moreover, the increase in the aliphatic chain length contributed to higher lipophilicity, with the **3Pd** complex being the most hydrophobic (LogP_o/w_ = 0.575 ± 0.033).

### 2.5. Antimicrobial Activity

#### 2.5.1. Antibacterial Activity

Upon confirming the stability of the complexes, in vitro antimicrobial activity of the palladium complexes **1Pd**–**3Pd** was evaluated against clinical isolates of Gram-positive bacteria (*Staphylococcus aureus*, *Listeria monocytogenes*, and *Enterococcus faecalis*) and Gram-negative bacteria (*Escherichia coli*, *Klebsiella pneumoniae*, *Pseudomonas aeruginosa*, *Proteus mirabilis*, and *Acinetobacter baumannii*), through the determination of the minimum inhibitory concentration (MIC) using the broth microdilution method. All complexes were tested at an initial (stock) concentration of 10 µM, whereas gentamicin, used as a positive control, was prepared from a stock solution of 52 µM (0.016 mg/mL).

The palladium complexes exhibited higher activity against Gram-positive strains (Table 3) than against Gram-negative strains (Table 4). MIC values for Gram-positive bacteria were relatively low, with the **3Pd** complex showing the most notable activity against *L. monocytogenes* (1.17 µg/mL) and *E. faecalis* (4.68 µg/mL). Moreover, it is worth noting that these complexes display superior antibacterial activity against these strains compared to other previously reported Pd(II) complexes [51,52]. These values were comparable and even lower than those observed for the positive control (gentamicin). Furthermore, considering that the complexes were evaluated at a substantially lower concentration than the control—approximately fivefold lower on a molar basis—this highlights a favorable activity/concentration relationship and therefore a remarkable inhibitory potential of the synthesized complexes. On the other hand, the **1Pd** complex showed higher activity against *S. aureus* (9.37 µg/mL) (Table 3). These results suggest that the characteristic cell wall of Gram-positive bacteria, which is primarily composed of a thick peptidoglycan layer, does not represent a significant barrier to the penetration of these metal complexes.

Regarding Gram-negative bacteria, the complexes exhibited inhibitory activity in the range of 9.37–37.5 µg/mL, depending on the microorganism (Table 4). For example, against *E. coli*, MIC values of 37.5 µg/mL for **1Pd**-**2Pd** and 15.75 µg/mL for **3Pd** were observed, once again indicating a relatively higher efficacy of the **3Pd** complex. It is worth highlighting that, even though the complexes were tested at lower concentrations than the positive control, they maintained a relevant biological response. These complexes have been shown to possess greater bioactivity against *E. coli* compared to previously reported Pd(II) complexes used against this strain [53,54]. Similarly, the complexes demonstrated considerable activity against *P. aeruginosa*, exhibiting MIC values in the range of 4.68–9.37 µg/mL.

#### 2.5.2. Antifungal Activity

The in vitro antifungal activity of complexes **1Pd**–**3Pd** was evaluated against three *Candida* species: *Candida albicans* (*C. albicans*), *Candida tropicalis* (*C. tropicalis*), and *Candida glabrata* (*C. glabrata*), using fluconazole as a reference compound. The assays were carried out from stock solutions of 10 µM for the complexes and 6530 µM (2 mg/mL) for the reference drug, from which the corresponding dilutions were prepared for the determination of the minimum fungicidal concentration (MFC). The obtained results demonstrate that all complexes exhibit significant activity at low concentration ranges (18.75–37.5 µg/mL), indicating a notable antifungal potential (Table 5). Against *Candida albicans*, all three complexes displayed fungicidal activity at 18.75 µg/mL, indicating high efficacy and comparable behavior among them, as well as greater activity than other Pd(II) complexes previously evaluated against this fungal strain [55]. In the case of *Candida tropicalis*, differences in the type of activity were observed: while **1Pd** exhibited a fungistatic effect at 37.5 µg/mL, both **2Pd** and **3Pd** showed fungicidal activity, with **3Pd** being the most active compound, achieving this effect at a lower concentration (18.75 µg/mL). Additionally, considering that the complexes were evaluated at a substantially lower concentration compared to the reference antifungal drug fluconazole, approximately 650-fold lower in molar terms, this pronounced difference further emphasizes their strong antifungal performance.

In contrast, against *Candida glabrata*, the complexes exhibited a differentiated response. Complex **1Pd** showed fungistatic activity at 37.5 µg/mL, whereas **2Pd** maintained a fungistatic effect at a lower concentration (18.75 µg/mL). Notably, complex **3Pd** demonstrated fungicidal activity at 18.75 µg/mL, positioning it as the most potent compound within the series. To date, this represents the first report on the antifungal activity of Pd(II) complexes, particularly those involving P^N-type ligands, against *C. tropicalis* and *C. glabrata* strains. As a reference control, fluconazole was evaluated, against which the tested strains exhibited resistance or limited susceptibility, showing activity only against *Candida albicans* at a significantly higher concentration (150 µg/mL). In contrast, the synthesized complexes displayed antifungal activity at substantially lower concentrations, highlighting their potential as alternative agents against resistant fungal strains.

Overall, these results indicate that slight structural variations in the complexes significantly influence their antifungal activity, with complex **3Pd** emerging as the most promising candidate due to its consistent fungicidal effect across all tested strains at lower concentrations.

These findings further indicate that the metal complexes exhibit antimicrobial activity within a lower concentration range than the reference drugs. This suggests that palladium complexes may exert antimicrobial and antifungal effects through mechanisms of action different from those of conventional drugs, potentially enabling them to overcome certain resistance mechanisms present in clinical strains. Furthermore, the variation observed among the evaluated complexes suggests that structural differences in P^N ligands may influence biological activity, possibly through changes in properties such as lipophilicity, complex stability, or the ability to interact with bacterial biomolecules. In this context, complex **3Pd** may be considered the most promising candidate, given its consistent bactericidal and fungicidal effects against all tested strains at lower concentrations.

### 2.6. Molecular Docking

**Figure 10:**
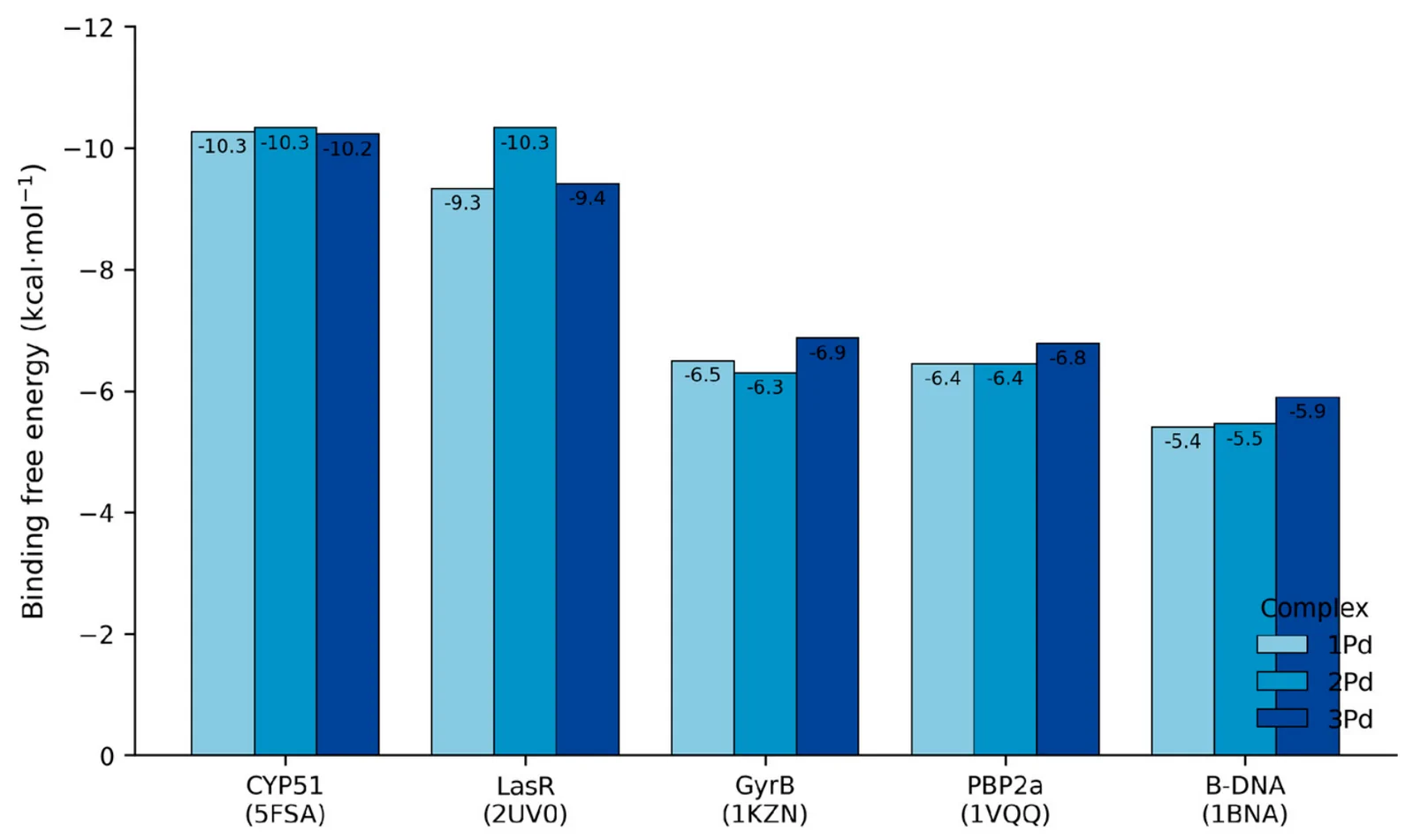
Predicted binding free energies (kcal/mol) of complexes **1Pd**–**3Pd** across the four molecular targets. More negative values denote stronger binding; the antifungal target CYP51 (5FSA) shows the most favorable interactions.

To provide a molecular-level rationale for the antimicrobial profile of complexes **1Pd**–**3Pd**, the relaxed structures were docked into four putative macromolecular targets representative of the assayed microorganisms; the resulting binding free energies are summarized in Table 6 and Figure 10. The complete cluster analysis of every complex–target pair is provided in Table S4.

**Figure 11:**
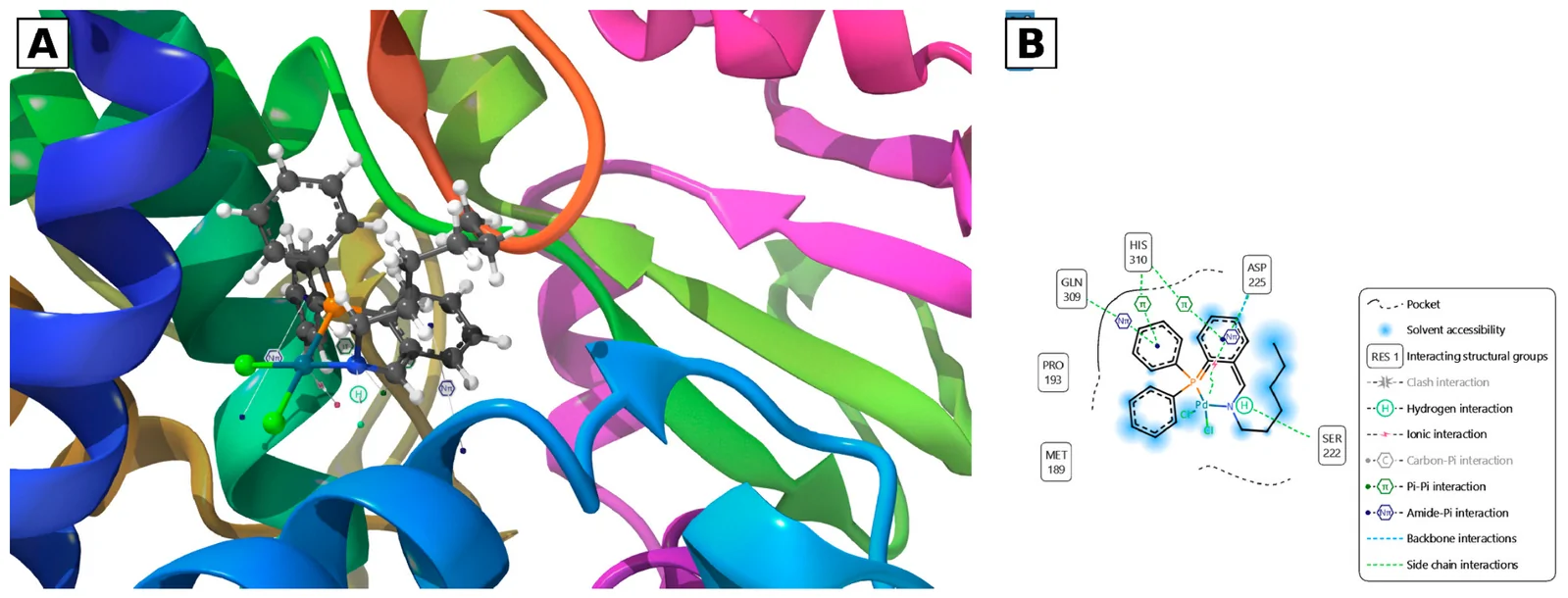
Binding pose of complex **3Pd** in *C. albicans* CYP51 (PDB 5FSA), rendered in SAMSON 2026 R1. (**A**) Three-dimensional view of **3Pd** within the enzyme pocket (protein as rainbow cartoon; complex as ball-and-stick with the Pd center and chloride ligands); (**B**) two-dimensional interaction diagram showing π-π and amide-π interactions of the phosphine phenyl rings with His310 and Gln309, hydrogen-bond and backbone contacts with Asp225, a side-chain hydrogen interaction with Ser222, and the hydrophobic pocket residues Pro193 and Met189.

**Figure 12:**
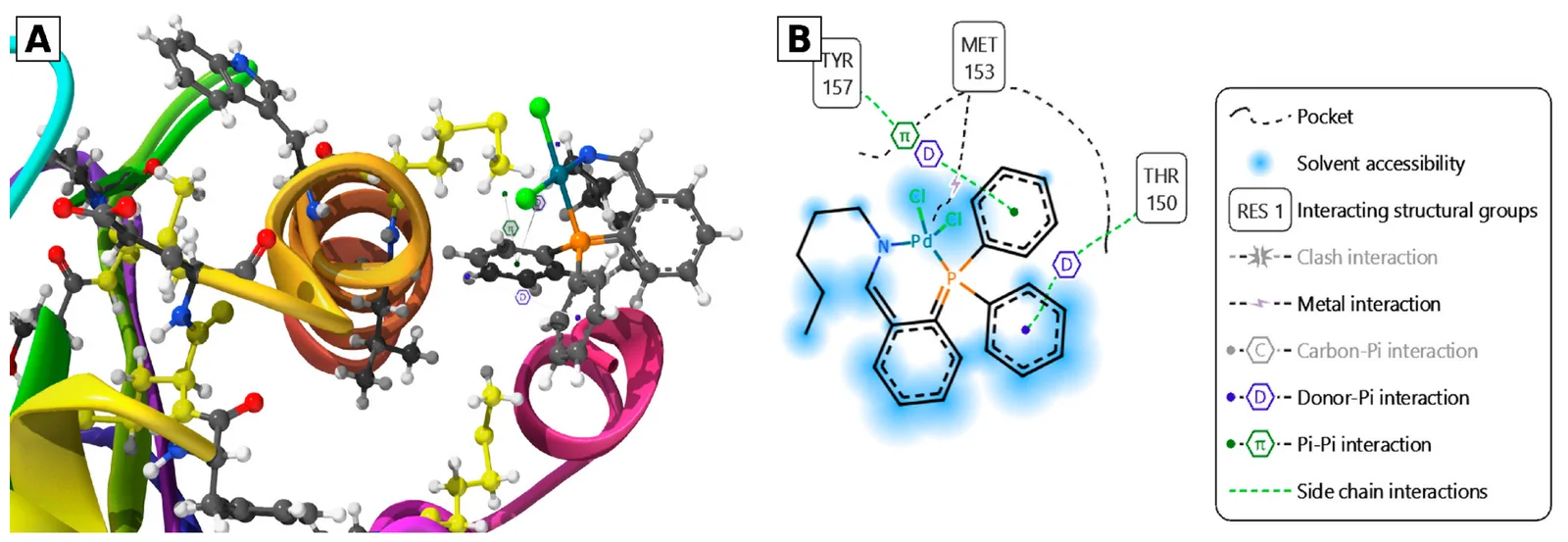
Binding pose of complex **2Pd** in P. aeruginosa LasR (PDB 2UV0), rendered in SAMSON 2026 R1. (**A**) Three-dimensional view of **2Pd** within the autoinducer-binding pocket (protein as cartoon; complex as ball-and-stick); (**B**) two-dimensional interaction diagram showing a metal interaction of the Pd-Cl unit with Met153, a hydrogen-bond and side-chain contact with Tyr157, and donor-π and π-π interactions involving Thr150 and the phosphine phenyl rings.

The most favorable interactions of the entire series were obtained for the fungal target sterol 14α-demethylase (CYP51), with binding free energies of −10.36, −10.63 and −10.75 kcal mol-1 for **1Pd**, **2Pd** and **3Pd** (Figure 11 and Figure 12), respectively; the lowest-energy pose was reproduced by 33–43 of the 50 independent runs in each case. The three complexes occupied the same region of the enzyme, delimited by Tyr118, Phe126, Ile131, Lys143 and Phe228, by the Gly303-Thr311 segment, and by the Pro462-Gly472 loop. The near-identical values across the series are consistent with the experimental antifungal data, in which the three complexes behaved as equipotent agents against *Candida albicans* (18.75 µg/mL), and point to interference with the ergosterol-biosynthesis pathway as a plausible contributor to the antifungal action of the complexes.

Against the canonical antibacterial targets DNA gyrase B (GyrB) and penicillin-binding protein 2a (PBP2a), the complexes showed moderate binding free energies (−5.96 to −6.38 and −6.54 to −7.12 kcal/mol, respectively). For GyrB, the poses occupied the ATPase site, contacting Val93, Leu94 and His95 as well as the Gly117-Val122 segment, although the search was less converged for this target (4–9 of 50 runs in the top-ranked cluster). For PBP2a, the search converged almost completely (46–49 of 50 runs, 2–4 distinct clusters), with the complexes occupying the transpeptidase region delimited by Tyr344, Thr398, Thr399 and Ser400. These values are compatible with the possible involvement of cell-division and cell-wall-biosynthesis machineries in the response. The weakest interactions of the series corresponded to the B-DNA dodecamer (−5.65 to −5.89 kcal/mol); for this target, the conformational search did not converge, with the 100 independent runs producing between 35 and 49 distinct clusters with no common binding mode among the three complexes, which is consistent with a weak, non-specific association with the duplex rather than with a defined binding site. Direct DNA binding is therefore unlikely to be a determinant of activity.

Across the three protein targets, the most lipophilic complex, **3Pd** (LogP*o*/*w* = 0.575), consistently gave the most favorable predicted binding energy, in line with its superior experimental activity, most notably against *L. monocytogenes* (MIC = 1.17 µg/mL) and *E. faecalis* (MIC = 4.68 µg/mL). This trend should nevertheless be interpreted with caution: the differences between complexes for a given target (0.39–0.58 kcal/mol) remain below the intrinsic uncertainty of the AutoDock4 scoring function, and the correspondence with the biological data is not uniform, since **1Pd** is the most active complex against *S. aureus* although **3Pd** is the best-scoring ligand for PBP2a. Accordingly, no structure–activity relationship is derived here from the computed energies. It must be stressed that molecular docking provides only a static, approximate ranking of relative affinities; the targets discussed above should be regarded as putative, and their involvement in the antimicrobial mechanism of complexes **1Pd**–**3Pd** requires validation through dedicated enzymatic and cell-based assays.

## 3. Materials and Methods

All reagents and solvents were commercially obtained from Sigma-Aldrich (St. Louis, MO, USA) and used without further purification. Melting points were measured on a MELT-TEMP II apparatus and are reported uncorrected. FT-IR spectra were recorded on a Thermo Fisher Scientific NICOLET IS-50 FT-IR spectrometer (Waltham, MA, USA) using the attenuated total reflectance (ATR) technique. UV-Vis spectra were obtained with an Agilent 8453 diode array spectrophotometer (Waldbronn, Germany). ^1^H, ^13^C{^1^H}, and ^31^P{^1^H} NMR spectra were recorded on a Bruker Avance III HD 700 MHz spectrometer (Rheinstetten, Germany). Chemical shifts are reported in ppm relative to TMS, using the residual solvent signals (DMSO-d_6_) as internal standard. Fast atom bombardment mass spectra (FAB^+^) were recorded on a The MStation JMS-700 spectrometer (Tokyo, Japan). Elemental analyses were performed on a Thermo Scientific Flash 2000 elemental analyzer using a Mettler Toledo XP6 Automated-S microbalance, (Greifensee, Switzerland) with sulfanilamide as standard (Thermo Scientific BN 217826 (Waltham, MA, USA); obtained values: *N* = 16.40%, *C* = 41.91%, *H* = 4.65%, *S* = 18.63%; certified values: *N* = 16.26%, *C* = 41.81%, *H* = 4.71%, *S* = 18.62%).

### 3.1. General Procedure for the Synthesis of P^N-Pd(II) Complexes with Difference Aliphatic Amines

All palladium complexes were synthesized following a procedure analogous to that previously reported by our research group [29], with slight modifications, including the in situ generation of all complexes. To a solution of 2-diphenylphosphinobenzaldehyde in dichloromethane, the corresponding aliphatic amines were added in a 1:1 molar ratio. The reaction mixture was refluxed for 24 h. Afterwards, the mixture was allowed to cool at room temperature, and the metal precursor [PdCl_2_(cod)] was added, followed by stirring at room temperature for 24 h. The reaction mixture was then concentrated to approximately 3 mL, and an excess of cold hexane was added, affording a yellow precipitate in all cases. The solid was collected by filtration, washed with cold hexane (3 × 10 mL), and dried under vacuum.

#### 3.1.1. Synthesis of **1Pd**

For **1Pd**: Yield: 57%. M.p. 196–197 °C. NMR de ^1^H (700 MHz, DMSO-d_6_, ppm): δ 8.59 (s, 1H, -CH=N), 7.99 (dd, cH, CH_Ar_, ^3^*J*_H-H_ = 7.0 Hz), 7.92 (t, dH, CH_Ar_, ^3^*J*_H-H_ = 7.0 Hz), 7.76 (t, eH, CH_Ar_, ^3^*J*_H-H_ = 7.0 Hz), 7.67 (t, jH, CH_Ar_, ^3^*J*_H-H_ = 7.0 Hz), 7.57 (td, hH, CH_Ar_, ^3^*J*_H-H_ = 7.0 Hz, ^4^*J*_H-H_ = 2.94 Hz), 7.38 (m, iH, CH_Ar_), 6.93 (td, fH, CH_Ar_, ^3^*J*_H-H_ = 7.0 Hz, ^4^*J*_H-H_ = 2.38 Hz), 4.26 (t, 2H, -CH_2_-, ^3^*J*_H-H_ = 7.0 Hz), 1.51 (p, 3H, -CH_2_-, ^3^*J*_H-H_ = 7.0 Hz), 0.76 (sx, 4H, -CH_2_-, ^3^*J*_H-H_ = 7.0 Hz), 0.64 (t, 5H, -CH_3_, ^3^*J*_H-H_ = 7.0 Hz). ^13^C{^1^H} NMR (175 MHz, DMSO-d_6_): δ 165.3 (s, 1C, -C=N), 136.7 (d, bC, C_Ar_, ^2^*J*_C-P_ = 15.7 Hz), 136.4 (d, cC, C_Ar_, ^3^*J*_C-P_ = 8.7 Hz), 134.2 (d, eC, C_Ar_, ^3^*J*_C-P_ = 7.0 Hz), 133.6 (complex, iC, C_Ar_), 133.5 (complex, fC, C_Ar_), 133.3 (s, dC, C_Ar_), 132.2 (s, jC, C_Ar_), 129.0 (d, hC, C_Ar_, ^2^*J*_C-P_ = 12.2 Hz), 125.4 (d, gC, C_Ar_, ^1^*J*_P-C_ = 59.5 Hz), 119.5 (d, aC, C_Ar_, ^1^J_P-C_ = 49.0 Hz), 65.1 (s, 2C, -CH_2_-), 32.3 (s, 3C, -CH_2_-), 18.8 (s, 4C, -CH_2_-), 13.5 (s, 5C, -CH_3_). ^31^P{^1^H} NMR (121 MHz, DMSO-d_6_): δ 31.2. MS-FAB^+^, *m*/*z* (%): 486 (100) [M^+^ – Cl^−^]. IR (ATR, υ(cm^−1^)) 1632 (C=N). Elem. Anal. Calc. For C_23_H_24_Cl_2_NPPd (522.75 g mol^−1^): C 52.85, H 4.63, N 2.68; found C 52.34, H 4.67, N 2.91.

#### 3.1.2. Synthesis of **2Pd**

For **2Pd**: Yield: 67%. M.p. 220–221 °C. NMR de ^1^H (700 MHz, DMSO-d_6_, ppm): δ 8.59 (s, 1H, -CH=N), 7.99 (dd, cH, CH_Ar_, ^3^*J*_H-H_ = 7.0 Hz), 7.91 (t, dH, CH_Ar_, ^3^*J*_H-H_ = 7.0 Hz), 7.74 (t, eH, CH_Ar_, ^3^*J*_H-H_ = 7.0 Hz), 7.66 (t, jH, CH_Ar_, ^3^*J*_H-H_ = 7.0 Hz), 7.57 (td, hH, CH_Ar_, ^3^*J*_H-H_ = 7.0 Hz, ^4^*J*_H-H_ = 2.8 Hz), 7.38 (m, iH, CH_Ar_), 6.93 (td, fH, CH_Ar_, ^3^*J*_H-H_ = 7.0 Hz, ^4^*J*_H-H_ = 3.7 Hz), 4.25 (t, 2H, -CH_2_-, ^3^*J*_H-H_ = 7.0 Hz), 1.52 (p, 3H, -CH_2_-, ^3^*J*_H-H_ = 7.0 Hz), 1.04 (sx, 4H, -CH_2_-, ^3^*J*_H-H_ = 7.0 Hz), 0.74 (p, 5H, -CH_2_-, ^3^*J*_H-H_ = 7.0 Hz), 0.66 (t, 6H, -CH_3_, ^3^*J*_H-H_ = 7.0 Hz). ^13^C{^1^H} NMR (175 MHz, DMSO-d_6_): δ 165.2 (s, 1C, -C=N), 136.7 (d, bC, C_Ar_, ^2^*J*_P-C_ = 15.7 Hz), 136.4 (d, cC, C_Ar_, ^3^*J*_C-P_ = 8.7 Hz), 134.2 (d, eC, C_Ar_, ^3^*J*_C-P_ = 7.0 Hz), 133.5 (complex, iC, C_Ar_), 133.5 (complex, fC, C_Ar_), 133.3 (s, dC, C_Ar_), 132.3 (s, jC, C_Ar_), 129.0 (d, hC, C_Ar_, ^2^*J*_C-P_ = 10.5 Hz), 125.5 (d, gC, C_Ar_, ^1^*J*_P-C_ = 59.5 Hz), 119.5 (d, aC, C_Ar_, ^1^*J*_C-P_ = 50.7 Hz), 65.5 (s, 2C, -CH_2_-), 27.7 (s, 3C, -CH_2_-), 27.7 (s, 4C, -CH_2_-), 21.7 (s, 5C, -CH_2_-), 13.6 (s, 6C, -CH_3_). ^31^P{^1^H} NMR (121 MHz, DMSO-d_6_): δ 31.4. MS-FAB^+^, *m*/*z* (%): 502 (100) [M^+^ - Cl^−^]. IR (ATR, υ(cm^−1^)): 1631 (C=N). Elem. Anal. Calc. For C_24_H_26_Cl_2_NPPd (536.77 g mol^−1^): C 53.70, H 4.88, N 2.61; found C 52.77, H 4.90, N 2.88.

#### 3.1.3. Synthesis of **3Pd**

For **3Pd**: Yield: 67%. M.p. 230–232 °C. NMR de ^1^H (700 MHz, DMSO-d_6_, ppm): δ 8.57 (s, 1H, -CH=N), 7.98 (dd, cH, CH_Ar_, ^3^*J*_H-H_ = 7.0 Hz), 7.90 (t, dH, CH_Ar_, ^3^*J*_H-H_ = 7.0 Hz), 7.75 (t, eH, CH_Ar_, ^3^*J*_H-H_ = 7.0 Hz), 7.66 (t, jH, CH_Ar_, ^3^*J*_H-H_ = 7.0 Hz), 7.56 (td, hH, CH_Ar_, ^3^*J*_H-H_ = 7.0 Hz, ^4^*J*_H-H_ = 3.0 Hz), 7.37 (m, iH, CH_Ar_), 6.92 (td, fH, CH_Ar_, ^3^*J*_H-H_ = 7.0 Hz, ^4^*J*_H-H_ = 3.22 Hz), 4.24 (t, 2H, -CH_2_-, ^3^*J*_H-H_ = 7.0 Hz), 1.51 (p, 3H, -CH_2_-, ^3^*J*_H-H_ = 7.0 Hz), 1.01 (m, 4H, 6H, -CH_2_CH_2_-), 0.74 (p, 5H, -CH_2_-, ^3^*J*_H-H_ = 7.0 Hz), 0.76 (m, 5H, 7H, -CH_2_CH_3_). ^13^C{^1^H} NMR (175 MHz, DMSO-d_6_): δ 165.3 (s, 1C, -C=N), 136.9 (d, bC, C_Ar_, ^2^*J*_P-C_ = 15.7 Hz), 136.5 (d, cC, C_Ar_, ^3^*J*_C-P_ = 8.7 Hz), 134.4 (d, eC, C_Ar_, ^3^*J*_C-P_ = 7.0 Hz), 133.7 (complex, iC, C_Ar_), 133.6 (complex, fC, C_Ar_), 133.5 (s, dC, C_Ar_), 132.5 (s, jC, C_Ar_), 129.2 (d, hC, C_Ar_, ^2^*J*_C-P_ = 12.2 Hz), 125.6 (d, gC, C_Ar_, ^1^*J*_C-P_ = 59.5 Hz), 119.6 (d, aC, C_Ar_, ^1^*J*_P-C_ = 50.7 Hz), 65.6 (s, 2C, -CH_2_-), 30.89 (s, 3C, -CH_2_-), 30.4 (s, 4C, -CH_2_-), 25.41 (s, 5C, -CH_2_-), 21.9 (s, 6C, -CH_2_-) 13.9 (s, 6C, -CH_3_). ^31^P{^1^H} NMR (121 MHz, DMSO-d_6_): δ 31.2. MS-FAB^+^, *m*/*z* (%): 516 (100) [M^+^ − Cl^−^]. IR (ATR, υ(cm^−1^)): 1631 (C=N). Elem. Anal. Calc. For C_25_H_28_Cl_2_NPPd (550.80 g mol^−1^): C 54.52, H 5.12, N 2.54; found C 53.78, H 5.16, N 2.94.

### 3.2. Stability Assay

Stock solutions of all complexes (0.1 M) were prepared in DMSO, and aliquots were taken to prepare a 2.5 × 10^−5^ M solution in a total volume of 2.0 mL. For aqueous solutions, 0.1% *v*/*v* of the stock solution was used, and the final volume was adjusted with Milli-Q water to 2.5 × 10^−5^ M. Subsequently, eight measurements were carried out at 0, 1, 2, 4, 8, 12, 24, and 48 h using an Agilent 8453 diode array UV-Vis spectrophotometer (Waldbronn, Germany).

### 3.3. Measure of LogP_o/w_

The octanol–water partition coefficients (K_o/w_) and LogP values of complexes **1Pd**–**3Pd** were determined using the “shaken-flask” method, as previously described [56]. Stock solutions of the studied compounds were prepared in n-octanol (4.0 mL; 2.5 µM for **1Pd**–**3Pd**) from a 0.01 M DMSO stock solution of the complexes. In centrifuge tubes, equal volumes of n-octanol and Milli-Q water were mixed and centrifuged at 7800 rpm for 5 min to separate the phases, which were then allowed to equilibrate for 24 h. Aliquots of the n-octanol phase were taken to prepare the complex stock solutions, which were vortexed for 5 min, and 2 mL of each solution was reserved in a separate centrifuge tube. To the remaining 2 mL, 2 mL of n-octanol-saturated Milli-Q water was added, and the mixtures were vigorously vortexed for 15 min. The octanol/water mixtures were then centrifuged at 7800 rpm for 5 min and allowed to stand for 24 h. UV-Vis absorption spectra of the n-octanol phases, as well as the reserved aliquots, were recorded using an Agilent 8453 diode array UV-Vis spectrophotometer. LogP_o/w_ values were calculated using Equation (1).

(1) Log P=Log(Ko/w)= Log AAo−A

where A_o_ is the absorbance of the reserved aliquots of the complexes at their respective maximum absorption wavelengths (λ_Abs_ (**1Pd**) = 233 nm, λ_Abs_ (**2Pd**) = 233 nm, and λ_Abs_ (**3Pd**) = 223 nm), and A is the absorbance of the n-octanol phase of the corresponding n-octanol/water mixtures at the same wavelengths.

### 3.4. Antibacterial Activity

The antibacterial activity of the metal complexes (**1Pd**–**3Pd**) was evaluated against a total of nine clinically relevant bacterial strains, including three Gram-positive and six Gram-negative species. Resistant strains were obtained from clinical isolates: *Staphylococcus aureus*, *Listeria monocytogenes*, and *Enterococcus faecalis* (Gram-positive), as well as *Escherichia coli*, *Klebsiella pneumoniae*, *Proteus* spp., *Acinetobacter baumannii*, *Pseudomonas aeruginosa*, and *Salmonella* spp. (Gram-negative). All strains were preserved and provided by the strain collection of the Universidad de Sonora.

Prior to the assays, bacteria were reactivated in Brain Heart Infusion (BHI) broth and subsequently resuspended in sterile saline to obtain a bacterial inoculum with a final density of approximately 1 × 10^8^ CFU/mL.

#### 3.4.1. Determination of the Minimum Inhibitory Concentration (MIC)

The minimum inhibitory concentrations (MICs) of the complexes were determined using the broth microdilution method, following the guidelines established by the Clinical and Laboratory Standards Institute [57,58]. Serial dilutions of the evaluated complexes, as well as the reference antibiotic gentamicin (0.312–10 µg/mL), were prepared in 96-well microtiter plates. The bacterial inoculum was prepared from metabolically active cultures (18–24 h) grown in BHI broth and adjusted to a turbidity equivalent to the 0.5 McFarland standard. Subsequently, 10 µL of the standardized inoculum was added to each well containing the pre-prepared serial dilutions of the complexes and the control. Plates were incubated at 37 °C for 18–24 h. The MIC was defined as the lowest concentration of the compound capable of visibly inhibiting bacterial growth, as assessed by the absence of turbidity in the wells. All assays were performed in triplicate. All MIC determinations were performed in triplicate for each bacterial strain, using three technical replicates under the same experimental conditions.

#### 3.4.2. Determination of the Minimum Bactericidal Concentration (MBC)

For the determination of the minimum bactericidal concentration (MBC), aliquots from the wells corresponding to concentrations equal to the MIC were plated onto Mueller–Hinton agar. Plates were incubated at 37 °C for 18–24 h, and the presence or absence of bacterial colonies was evaluated. The MBC was defined as the lowest concentration of the compound capable of completely inhibiting bacterial growth, allowing differentiation between bacteriostatic and bactericidal effects.

### 3.5. Antifungal Activity

The antifungal activity of the metal complexes was evaluated against three clinically relevant yeast strains: *Candida albicans*, *Candida tropicalis*, and *Candida glabrata*. These strains were obtained from the strain collection of the Universidad de Sonora and preserved under standard laboratory conditions.

Prior to the assays, the yeasts were reactivated in YEPD (yeast extract peptone dextrose) broth containing yeast extract (10%), peptone (20%) and dextrose (20%) and incubated for 24 h at 30 °C. The cultures were subsequently resuspended in sterile saline solution to obtain a yeast inoculum with a final density of approximately 1 × 10^6^ CFU/mL. The antifungal activity of the compounds was evaluated by the broth microdilution method in YEPD medium, using Fluconazole as a reference control. The minimum inhibitory concentration (MIC) was defined as the lowest concentration of the complexes that prevented visible yeast growth after incubation.

#### 3.5.1. Determination of the Minimum Inhibitory Concentration (MIC)

The MIC values of the complexes were determined using the broth microdilution method, following the general guidelines established by the Clinical and Laboratory Standards Institute (CLSI). Serial dilutions of the evaluated complexes were prepared in 96-well microplates using YEPD medium.

The yeast inoculum was obtained from metabolically active cultures (24 h) grown in YEPD broth and adjusted to a turbidity equivalent to the 0.5 McFarland standard (approximately 1–5 × 10^6^ CFU/mL). Subsequently, 10 µL of the standardized inoculum were added to each well containing the serial dilutions of the complexes and the corresponding controls, including Fluconazole. The plates were incubated at 30 °C for 24–48 h.

The MIC was defined as the lowest concentration of each compound capable of visibly inhibiting yeast growth (*Candida albicans*, *Candida tropicalis*, and *Candida glabrata*), as determined by the absence of turbidity in the wells [57,58]. All assays were performed in triplicate.

#### 3.5.2. Determination of the Minimum Fungicidal Concentration (MFC)

To determine the minimum fungicidal concentration (MFC), aliquots from wells corresponding to concentrations equal to the MIC were subcultured onto Sabouraud dextrose agar plates. The plates were incubated at 30 °C for 24–48 h, after which the presence or absence of yeast colonies was evaluated.

The MFC was defined as the lowest concentration of the compound that completely prevented yeast growth (*Candida albicans*, *Candida tropicalis*, and *Candida glabrata*), allowing discrimination between fungistatic and fungicidal effects [57,58].

### 3.6. Data Collection and Refinement for Complex ***3Pd***

The crystal of the complex was obtained from a saturated solution in a DMSO/hexane mixture (1:1) and subsequently mounted on a Bruker Smart Apex II diffractometer (Karlsruhe, Germany) with a Mo Kα X-ray source (λ = 0.71073 Å). The detector was positioned 5.0 cm from the crystals, and frames were collected with a scan width of 0.5° in ω and an exposure time of 10 s per frame. Frames were integrated using the Bruker SAINT software package version 6.45 employing a narrow-frame integration algorithm. The structures were solved by Patterson methods using SHELXS-2014/7 [59]. Remaining atoms were located through a few cycles of least-squares refinement and difference Fourier maps. Hydrogen atoms were placed in calculated positions and allowed to “ride” on their parent atoms. Thermal parameters for hydrogen atoms in phenyl groups were refined using Ueq = 1.2 Å, the value of the attached atom. Final refinement cycles were performed on all non-zero data using SHELXL-2014/7 [59]. Absorption corrections were applied using the program SADABS [60].

### 3.7. Computational Details

Building on our group’s previous use of molecular docking to study the biomolecular interactions of metal complexes [61,62], a molecular docking study was performed against a panel of biologically relevant macromolecular targets of the assayed microorganisms (Table 7) to gain molecular-level insight into the antibacterial and antifungal activity of complexes **1Pd**–**3Pd** and to rationalize the structure–activity relationships derived from the in vitro assays. Since palladium is not parameterized in conventional docking force fields, the calculations were carried out with MetalDock, an open access workflow specifically designed for the reproducible docking of metal complexes, which couples a quantum-mechanical (QM) description of the organometallic fragment with the AutoDock4 docking tool [63,64].

#### 3.7.1. Geometry Optimization of the Pd(II) Complexes

The molecular geometries of complexes **1Pd**–**3Pd** were fully optimized within the framework of density functional theory (DFT) using the ORCA 6.0.1 program package [65,66]. The hybrid B3LYP exchange-correlation functional [67,68] was employed together with the all-electron DGDZVP basis set for every atom, including the palladium center [69]. Long-range dispersion was accounted for through Grimme’s atom-pairwise D3 correction with Becke–Johnson damping (D3BJ), which is particularly relevant given the extended aromatic surfaces of the diphenylphosphine moieties [70,71]. Each complex was treated as a neutral, closed-shell species (total charge = 0; singlet ground state, S = 0), consistent with a d^8^ Pd(II) ion in a square-planar coordination environment; the starting coordinates were taken from the single-crystal X-ray structure of **3Pd** and from analogous models of **1Pd** and **2Pd**. This level of theory is consistent with our previous density functional theory studies on transition-metal complexes [72]. The resulting geometries, together with the CM5 atomic partial charges derived from a Hirshfeld population analysis of the converged density, were used as input for the docking calculations. Cartesian coordinates, total energies and partial charges are collected in Tables S2 and S3.

#### 3.7.2. Selection and Preparation of the Molecular Targets

Macromolecular targets were selected to reflect both the antimicrobial spectrum of the complexes and the mechanisms of the reference drugs (Table 7). For the antifungal activity, the crystal structure of sterol 14α-demethylase (CYP51/ERG11) from *Candida albicans* (PDB ID 5FSA), the molecular target of the azole antifungals exemplified by the fluconazole reference, was selected [70]. For the antibacterial activity, the 24 kDa N-terminal ATPase domain of DNA gyrase subunit B from *Escherichia coli* (PDB ID 1KZN) was selected as a representative Gram-negative type II topoisomerase target [73], and penicillin-binding protein 2a (PBP2a) from *Staphylococcus aureus* (PDB ID 1VQQ) as a representative Gram-positive cell-wall-biosynthesis target [74]. The ligand-binding domain of the quorum-sensing regulator LasR from *Pseudomonas aeruginosa* (PDB ID 2UV0) was selected to probe the pronounced activity recorded against this pathogen [75]. Finally, the Drew–Dickerson B-DNA dodecamer (PDB ID 1BNA) was used to assess the propensity of the square-planar Pd(II) complexes to interact with nucleic acids [76]. All coordinate files were retrieved from the RCSB Protein Data Bank [77].

Before docking, each structure was curated by removing crystallographic water molecules and buffer or crystallization additives; alternate conformations were reduced to the highest-occupancy rotamer, and the hydrogen atoms present in the deposited models were removed. Because the automated receptor-preparation workflow could not parameterize the iron-containing heme cofactor, the heme group of CYP51 (5FSA) was removed before receptor preparation, and the grid box was instead centered on the validated heme/azole site; selenomethionine residues in the LasR structure (2UV0) were reverted to canonical methionine. A single representative chain was retained for the monomeric targets. Polar hydrogen atoms and Gasteiger partial charges were subsequently assigned during conversion of the receptors to the AutoDock PDBQT format [78,79], with protonation states corresponding to physiological pH (7.4).

#### 3.7.3. Molecular Docking

Docking of the DFT-optimized complexes **1Pd**–**3Pd** was conducted with MetalDock [63]. Within this protocol, the Lennard–Jones parameters required to describe the palladium atom, an element absent from the standard AutoDock force field, were supplied by the MetalDock parameter set, obtained through a Monte Carlo sampling scheme and shown to generalize across metal complexes, while the corresponding atomic partial charges were taken from the ORCA calculations (CM5 scheme), thereby ensuring a physically consistent treatment of the metal coordination sphere [79]. For each target, the search space was defined as a cubic grid box of 60 points per dimension with the default spacing of 0.375 Å, centered on the centroid of the co-crystallized ligand delineating the validated binding site—namely the azole/heme pocket of CYP51 (5FSA), the ATPase site of GyrB (1KZN) and the autoinducer pocket of LasR (2UV0). For the apo PBP2a structure (1VQQ), the box was centered on the catalytic Ser403 of the transpeptidase domain; in the B-DNA dodecamer (1BNA), it was enlarged to encompass the entire duplex in a blind-docking scheme. Conformational sampling was performed with the Lamarckian genetic algorithm implemented in AutoDock4, using random initial ligand placement [64,80]; the ten lowest-energy poses were retained for each complex–target pair and ranked according to their predicted binding free energy (Δ*G*, kcal/mol). The most favorable pose of the most populated cluster was selected as representative of the binding mode.

#### 3.7.4. Validation and Analysis of the Docking Protocol

The docking protocol was validated by redocking the native ligand co-crystallized with each target for which one is available, using the same receptor model and the same grid box employed for the metal complexes. The best pose reproduced the crystallographic conformation with a symmetry-corrected heavy-atom RMSD of 1.47 Å for clorobiocin in GyrB (1KZN), this being also the top-ranked solution, and of 2.46 Å for posaconazole in CYP51 (5FSA); the sampling settings and the number of poses generated are given in Table S5 and the superpositions in Figure S18. No co-crystallized ligand suitable for conventional redocking is available for PBP2a, whose deposited β-lactam complexes are covalent acyl-enzyme adducts, or for the B-DNA dodecamer, and these two systems are therefore reported as exploratory. For each complex–target pair, the poses generated by the independent genetic-algorithm runs were grouped by a ranked cluster analysis with a 2.0 Å RMSD tolerance, and the pose with the lowest predicted binding free energy was taken as representative of the binding mode; the complete cluster distributions are given in Table S3. The principal non-covalent contacts of each binding mode were identified and the two- and three-dimensional interaction diagrams visualized using SAMSON 2026 R1 [81]. The predicted energies are used here only to compare the relative behavior of the three complexes and are not interpreted quantitatively, since the differences between complexes for a given target are of the same order as the uncertainty of the AutoDock4 scoring function and the degree of convergence differs between targets (Table S4). Finally, the calculations refer to the intact neutral complexes and do not consider aquated or ligand-exchange species that might form in the culture medium.

## 4. Conclusions

A straightforward methodology has been developed for the synthesis of three new P^N-Pd(II) complexes, which were fully characterized. The molecular structure of complex **3Pd** was unequivocally determined by single-crystal X-ray diffraction, allowing for supramolecular studies on the interactions present within the crystal lattice. These analyses revealed that CH···π and CH···Cl interactions contribute most significantly to the stabilization of the crystal network. Through in vitro assays, antibacterial and antifungal studies were evaluated against Gram-positive and Gram-negative bacterial strains, as well as fungal strains of *Candida* species. In most of the strains tested, complex **3Pd** was the most active, exhibiting superior MIC and MFC values compared to its homologous complexes, as well as the reference drugs gentamicin and fluconazole, which could be attributed to the longer aliphatic chain present in its structure.

These experimental findings were complemented by molecular docking studies employing a quantum-mechanical description of the palladium center through the MetalDock/ORCA approach. All three complexes exhibited their most favorable predicted interactions with the fungal target sterol 14α-demethylase (CYP51; ΔG ≈ −10.4 to −10.8 kcal/mol), consistent with their uniform fungicidal activity against *Candida* species. Moderate binding affinities were obtained for DNA gyrase B and penicillin-binding protein 2a, consistent with the experimentally determined antibacterial activity, whereas the weak and non-convergent interaction with the B-DNA dodecamer indicates that direct binding to nucleic acids is unlikely to be a determinant of biological activity. Across the three protein targets, the most lipophilic complex, **3Pd**, displayed the most favorable predicted binding energy, mirroring its superior experimental performance in terms of MIC and MFC values, particularly against *Listeria monocytogenes* (MIC = 1.17 µg/mL). These differences nevertheless fall within the uncertainty of the scoring function, and the docking results are therefore presented as a qualitative, exploratory exercise that identifies putative macromolecular partners requiring experimental validation. Taken together, these findings identify **3Pd** as the most promising candidate of the present series, pending the evaluation of its cytotoxicity and selectivity.

Likewise, the P^N-Pd(II) complexes (**1Pd**–**3Pd**) are currently being evaluated as potential anticancer agents to assess and validate their cytotoxic effects against both cancerous and non-cancerous cell lines. The results of these studies will be reported in due course and are expected to contribute to a broader understanding of the diverse biological properties of these complexes.

## Figures and Tables

**Table 1:** Crystallographic data for compound **3Pd**.

	3Pd
Formula	C_25_H_28_Cl_2_NPPd
Formula weight	550.75
Temperature (K)	150(2)
Wavelength (Å)	0.71073
Crystal system	Monoclinic
Space group	P2_1_/n
*a* (Å)	9.9054(6)
*b* (Å)	21.1133(13)
*c* (Å)	11.4599(8)
*α* (°)	90
*β* (°)	91.152(3)
*γ* (°)	90
*V* (Å^3^)	2396.2(3)
*Z*	4
*δ_cal_* (g/cm^3^)	1.527
*μ* (mm^−1^)	1.077
*F*(000)	1120
Crystal size (mm^3^)	0.305 × 0.302 × 0.186
Theta range (°)	2.271 to 27.491
Reflections collected	69077
Data/restraints/parameters	5421/0/273
Goodness-of-fit	1.192
Final *R* indices [*I* > 2*σ(I)*]	*R*_1_ = 0.0208, w*R*_2_ = 0.0463
*R* indices (all data)	*R*_1_ = 0.0235, w*R*_2_ = 0.0483
Largest diff. peak and hole (e.Å^−3^)	0.354 and −0.510

**Table 2:** Main interactions in the molecular structure of complex **3Pd**.

Compound		(Å)	(Å)	(°)	
	Interaction	D-X···A	D···A	D-X···A	Symmetry Operation
**3Pd**	C4H4···C11	2.970	3.726(3)	137.5	1 − x, 1 − y, 1 + z
	C3H3···Cl0	2.767	3.564(3)	142.0	1 − x, 1 − y, 1 + z
	C9H9···C18	2.883	3.587(3)	131.9	1 − x, 1 − y, 1 + z
	C10H10···C17	2.901	3.733(3)	146.9	1 − x, 1 − y, 1 + z
	C22H22B···C5	2.955	3.871(3)	154.4	−1/2 + x, 1/2 − y, 1/2 + z
	C24H24A···C6	2.915	3.763(3)	144.2	−1/2 + x, 1/2 − y, 1/2 + z
	C4H4···Cl1	3.015	3.711(2)	131.4	−1 + x, y, z
	C3H3···[C14-C19]	3.055	3.810	137.5	1 − x, 1 − y, 1 − z
	C22H22A-[C-C13]	3.007	3.852	144.0	1 − x, 1−y, 1 − z

**Table 3:** Minimum inhibitory concentration (MIC) of complexes **1Pd**–**3Pd** against Gram-positive bacteria ^a^.

Compound	Gram-Positive Bacteria		
	*S. aureus*	*L. monocytogenes*	*E. faecalis*
**1Pd**	9.37 ^b^	4.68 ^b^	9.37 ^b^
**2Pd**	18.75 ^c^	2.34 ^b^	9.37 ^c^
**3Pd**	18.75 ^c^	1.17 ^c^	4.68 ^c^
Gentamicin	4.68 ^c^	1.17 ^c^	1.17 ^c^

^a^ Result expressed in µg/mL; ^b^ Bacteriostatic; ^c^ Bactericidal.

**Table 4:** Minimum inhibitory concentrations (MICs) of complexes **1Pd**–**3Pd** against Gram-negative bacteria ^a^.

Compound	Gram-Negative Bacteria					
	*E. coli*	*K. pneumoniae*	*P. mirabilis*	*A. baumannii*	*P. aeruginosa*	*S.* spp.
**1Pd**	37.5 ^b^	37.5 ^b^	37.5 ^b^	18.75 ^b^	9.37 ^c^	18.75 ^b^
**2Pd**	37.5 ^c^	37.5 ^b^	37.5 ^c^	18.75 ^c^	9.37 ^c^	9.37 ^c^
**3Pd**	18.75 ^c^	37.5 ^c^	18.75 ^c^	9.37 ^c^	4.68 ^c^	9.37 ^c^
Gentamicin	150 ^b^	150 ^b^	18.75 ^b^	4.68 ^c^	4.68 ^c^	9.37 ^b^

^a^ Result expressed in µg/mL; ^b^ Bacteriostatic; ^c^ Bactericidal.

**Table 5:** Minimum fungicidal concentrations (MFCs) determined for complexes **1Pd–3Pd** against different fungal species ^a^.

Compound	Fungi		
	*C. albicans*	*C. tropicalis*	*C. glabrata*
**1Pd**	18.75 ^b^	37.50 ^c^	37.50 ^c^
**2Pd**	18.75 ^b^	37.50 ^b^	18.75 ^c^
**3Pd**	18.75 ^b^	18.75 ^b^	18.75 ^b^
fluconazole	150 ^c^	-	-

^a^ Result expressed in µg/mL; ^b^ Fungicidal; ^c^ Fungistatic; - No inhibition.

**Table 6:** Binding free energies (kcal/mol) of complexes **1Pd**–**3Pd** against the selected targets.

Complex	CYP51 (5FSA)	GyrB (1KZN)	PBP2a (1VQQ)	B-DNA (1BNA)
1Pd	−10.36 (41/50)	−6.04 (4/50)	−6.54 (49/50)	−5.65 (1/100)
2Pd	−10.63 (43/50)	−5.96 (8/50)	−6.75 (47/50)	−5.89 (2/100)
3Pd	−10.75 (33/50)	−6.38 (9/50)	−7.12 (46/50)	−5.66 (1/100)

Lowest (most favorable) AutoDock4 binding free energy per complex–target pair, obtained from 50 independent Lamarckian genetic-algorithm runs (100 for the B-DNA dodecamer) on a grid box centered on the binding site. The number of runs in the top-ranked cluster is given in parentheses. More negative values indicate stronger predicted binding.

**Table 7:** Macromolecular targets selected for the molecular docking study of complexes **1Pd**–**3Pd**.

PDB ID	Molecular Target	Organism	Co-Crystallized Ligand (Binding Site)
5FSA	Sterol 14α-demethylase (CYP51/ERG11)	*C. albicans*	Posaconazole (azole/heme site)
1KZN	DNA gyrase B, 24 kDa domain	*E. coli*	Clorobiocin (ATPase site)
1VQQ	Penicillin-binding protein 2a (PBP2a)	*S. aureus*	apo (catalytic Ser403)
2UV0	LasR, ligand-binding domain	*P. aeruginosa*	3-oxo-C12-homoserine lactone
1BNA	B-DNA dodecamer (Drew–Dickerson)	synthetic	— (blind docking)

## Data Availability

The original contributions presented in this study are included in the article/Supplementary Materials. Further inquiries can be directed to the corresponding authors.

## Supplementary Materials

The following supporting information can be downloaded at: https://www.mdpi.com/article/10.3390/antibiotics15100968/s1, Table S1. Examples of the antibacterial activity of different Pd(II) complexes; Table S2. Cartesian coordinates, total energies and residual forces of the relaxed structures of complexes **1Pd**–**3Pd**; Table S3. CM5 atomic partial charges of the metal coordination sphere of complexes **1Pd**–**3Pd**; Table S4. Docking results and cluster analysis for complexes 1Pd–3Pd against the selected molecular targets; Table S5. Redocking validation of the docking protocol. Figure S1. ^1^H NMR of (**1Pd**) in DMSO-d_6_; Figure S2. ^13^C{^1^H} NMR of (**1Pd**) in DMSO-d_6_; Figure S3. ^31^P{^1^H} NMR of (**1Pd**) in DMSO-d_6_; Figure S4. ^1^H NMR of (**2Pd**) in DMSO-d_6_; Figure S5. ^13^C{^1^H} NMR of (**2Pd**) in DMSO-d_6_; Figure S6. ^31^P{^1^H} NMR of (**2Pd**) in DMSO-d_6_; Figure S7. ^1^H NMR of (**3Pd**) in DMSO-d_6_; Figure S8. ^13^C{^1^H} NMR of (**3Pd**) in DMSO-d_6_; Figure S9. ^31^P{^1^H} NMR of (**3Pd**) in DMSO-d_6_; Figure S10. FAB^+^-MS spectrum of (**1Pd**); Figure S11. FAB^+^-MS spectrum of (**2Pd**); Figure S12. FAB^+^-MS spectrum of (**3Pd**); Figure S13. IR spectra of (**1Pd**); Figure S14. IR spectra of (**2Pd**); Figure S15. IR spectra of (**3Pd**); Figure S16. Stability assays by UV-Vis of the **1Pd**: **A**) DMSO and **B**) with 0.1% (*v*/*v*) DMSO/H_2_O; Figure S17. Stability assays by UV-Vis of the **2Pd**: **A**) DMSO and **B**) with 0.1% (*v*/*v*) DMSO/H_2_O. The following are available online. CCDC 2581267 (**3Pd**) contains the supplementary crystallographic data for this paper. Figure S18. Superposition of the crystallographic conformation (blue) and the best docked pose (red) of the native ligand of each validated target. Coordinates are provided as 1KZN_superposition.pdb and 5FSA_superposition.pdb, in which chain A is the crystallographic conformation and chain B the docked pose. These data can be obtained free of charge via www.ccdc.cam.ac.uk/cgi-bin/catreq.cgi (accessed on 17 August 2026), by e-mailing data_request@ccdc.cam.ac.uk, or by contacting: The Cambridge Crystallographic Data Centre, 12 Union Road, Cambridge CB2 1EZ, UK, Fax: +44(0)-1223-336033.
