# Supplementary material for: Development of P^N-Pd(II) Type Complexes Derived from Aliphatic Amines as Potential Antibacterial and Antifungal Agents
Source: Antibiotics (Basel). 2026 Sep 30;15(10):968. doi: 10.3390/antibiotics15100968 (PMC13700911; doi:10.3390/antibiotics15100968)
Supplement: Supplementary file 1 [file antibiotics-15-00968-s001.zip › antibiotics-4537546-supplementary.pdf]

Supporting Information

Development of P^N-Pd(II) Type Complexes Derived from Aliphatic Amines as Potential Antibacterial and Antifungal Agents

Michelle Acosta-Encinas [1], Andrés Amaya-Flórez [2], Jordi R. Galindo [2], Simón Hernández-Ortega [2], Maritza Lizeth Alvarez-Ainza [3], Josue Juarez [4], Adrián L. Orjuela [5], Jorge Alí-Torres [6], Alberto Aragón-Muriel [7], Viviana Reyes-Márquez [3,*] and David Morales-Morales [2,*]

[1] Departamento de Investigación en Polímeros y Materiales, Universidad de Sonora, Luis Encinas y Rosales S/N, Hermosillo 83000, Sonora, Mexico; a217204227@unison.mx

[2] Instituto de Química, Universidad Nacional Autónoma de México, Circuito Exterior S/N, Ciudad de México 04510, Coyoacán, Mexico; andresamaya93@comunidad.unam.mx (A.A.-F.); jordi.ruiz@cch.unam.mx (J.R.G.); simonho@unam.mx (S.H.-O.)

[3] Departamento de Ciencias Químico-Biológicas, Universidad de Sonora, Luis Encinas y Rosales S/N, Hermosillo 8300, Sonora, Mexico; maritza.alvarez@unison.mx

[4] Departamento de Física, Universidad de Sonora, Luis Encinas y Rosales S/N, Hermosillo 83000, Sonora, Mexico; josue.juarez@unison.mx

[5] Plataforma de Biología y Química Computacionales. Instituto de Investigaciones Científicas y Servicios de Alta Tecnología (INDICASAT AIP), Panamá City 0843-01103, Panama; aorjuela@indicasat.org.pa

[6] Departamento de Química, Universidad Nacional de Colombia-Sede Bogotá, Bogotá 111321, Colombia; jialit@unal.edu.co

[7] Grupo de Investigación Bioquímicas (GIB), Departamento de Química, Universidad del Magdalena, Santa Marta 470004, Colombia; aaragonm@unimagdalena.edu.co

* Correspondence: viviana.reyes@unison.mx (V.R.-M.); damor@unam.mx (D.M.-M.)

Table of Contents

Table S1 Examples of the antibacterial activity of different Pd(II) complexes

Figure S1 ^{1}H NMR of (**1Pd**) in DMSO-d$_6$

Figure S2 ^{13}C{^{1}H} NMR of (**1Pd**) in DMSO-d$_6$

Figure S3 ^{31}P{^{1}H} NMR of (**1Pd**) in DMSO-d$_6$

Figure S4 ^{1}H NMR of (**2Pd**) in DMSO-d$_6$

Figure S5 ^{13}C{^{1}H} NMR of (**2Pd**) in DMSO-d$_6$

Figure S6 ^{31}P{^{1}H} NMR of (**2Pd**) in DMSO-d$_6$

Figure S7 ^{1}H NMR of (**3Pd**) in DMSO-d$_6$

Figure S8 ^{13}C{^{1}H} NMR of (**3Pd**) in DMSO-d$_6$

Figure S9 $^{31}P\{^{1}H\}$ NMR of (**3Pd**) in DMSO-d_6

Figure S10 FAB^{+}-MS spectrum of (**1Pd**)

Figure S11 FAB^{+}-MS spectrum of (**2Pd**)

Figure S12 FAB^{+}-MS spectrum of (**3Pd**)

Figure S13 IR spectra of (**1Pd**)

Figure S14 IR spectra of (**2Pd**)

Figure S15 IR spectra of (**3Pd**)

Figure S16 Stability assays of **1Pd**

Figure S17 Stability assays of **2Pd**

Figure S18 RMSD

Table S2 Cartesian coordinates

Table S3 CM5 atomic partial charges

Table S4 Docking results and clusters analysis

Table S5 Redocking validation of the docking protocol

Table S1. Examples of the antibacterial activity of different Pd(II) complexes

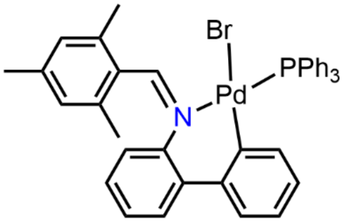 Albert and coworkers	Significant inhibition of cathepsin B

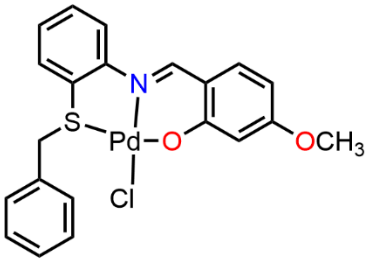 Barman and coworkers	Very good antibacterial activities against gram-positive and gram-negative bacteria
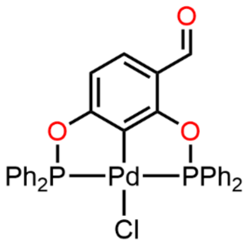 Morales-Morales and coworkers	Most active against *S. aeurus*

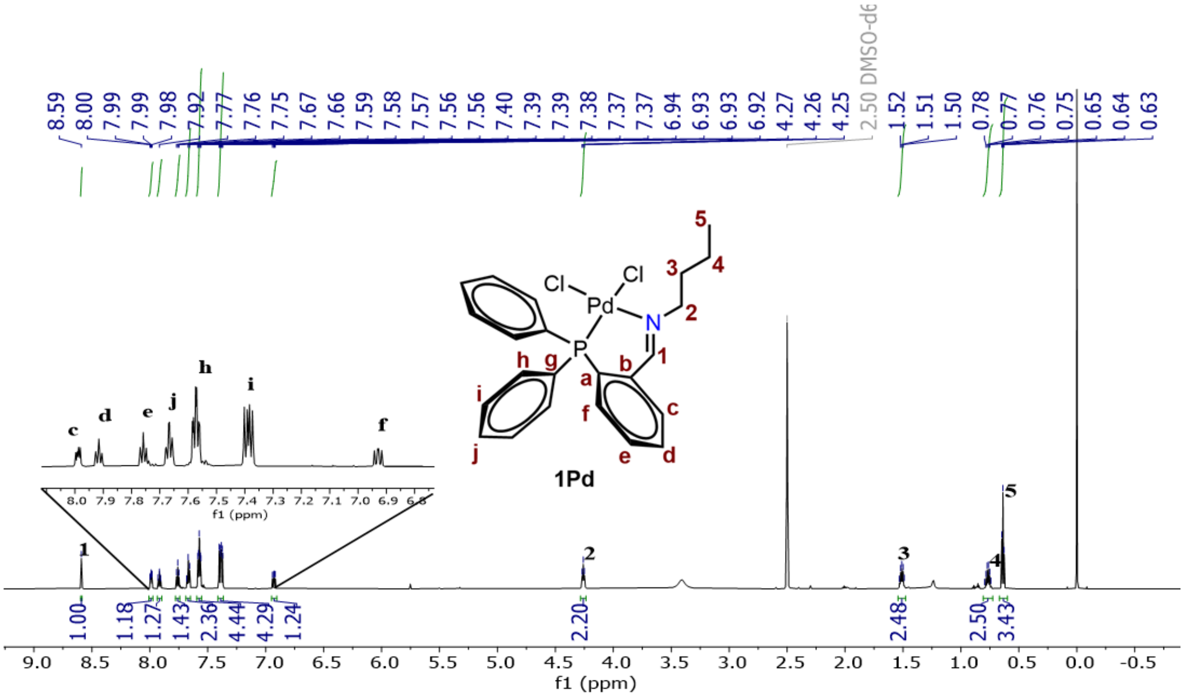

Figure S1. ^{1}H NMR of (**1Pd**) in DMSO-d$_6$.

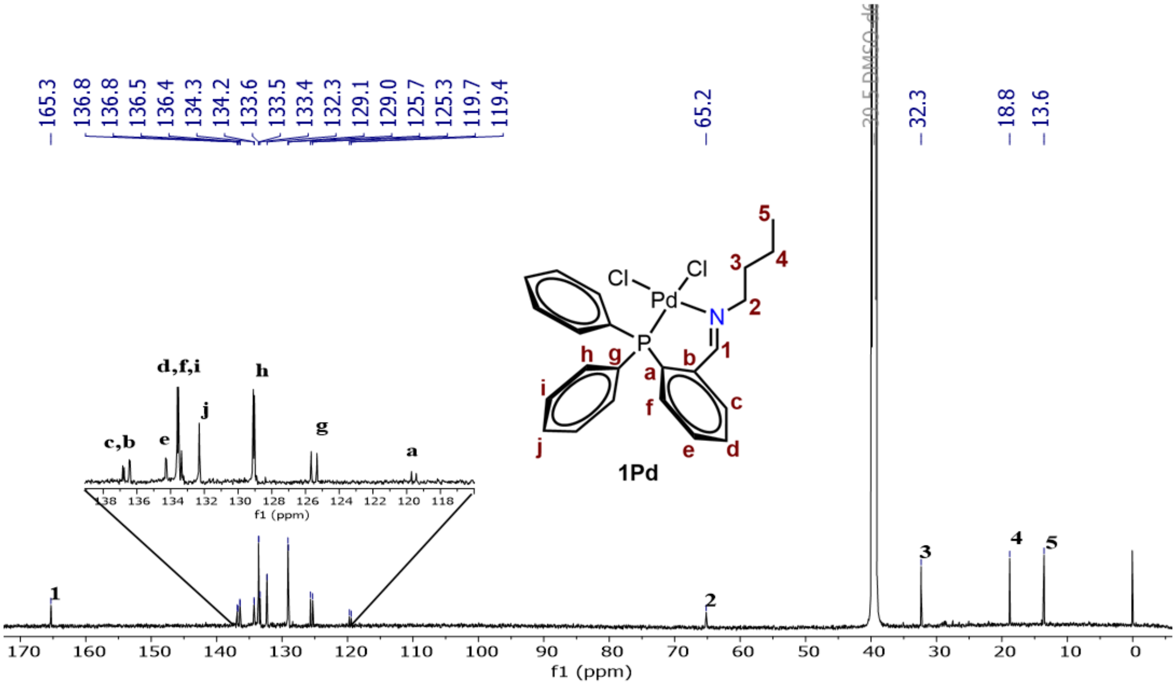

Figure S2. $^{13}C\{^{1}H\}$ NMR of (**1Pd**) in DMSO-d6.

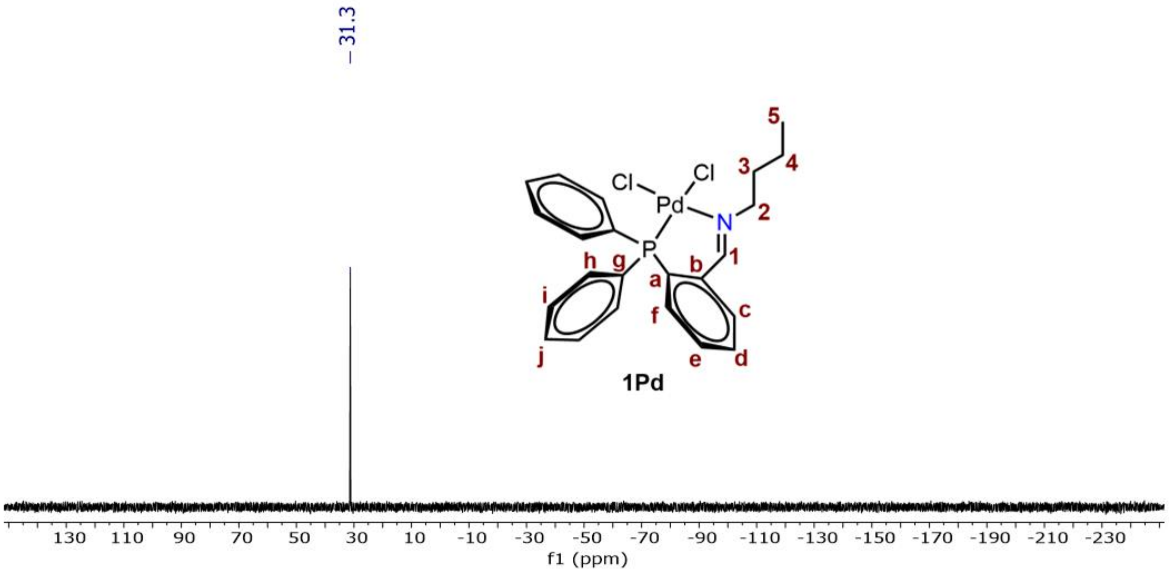

Figure S3. $^{31}P\{^{1}H\}$ NMR of (**1Pd**) in DMOS-d6.

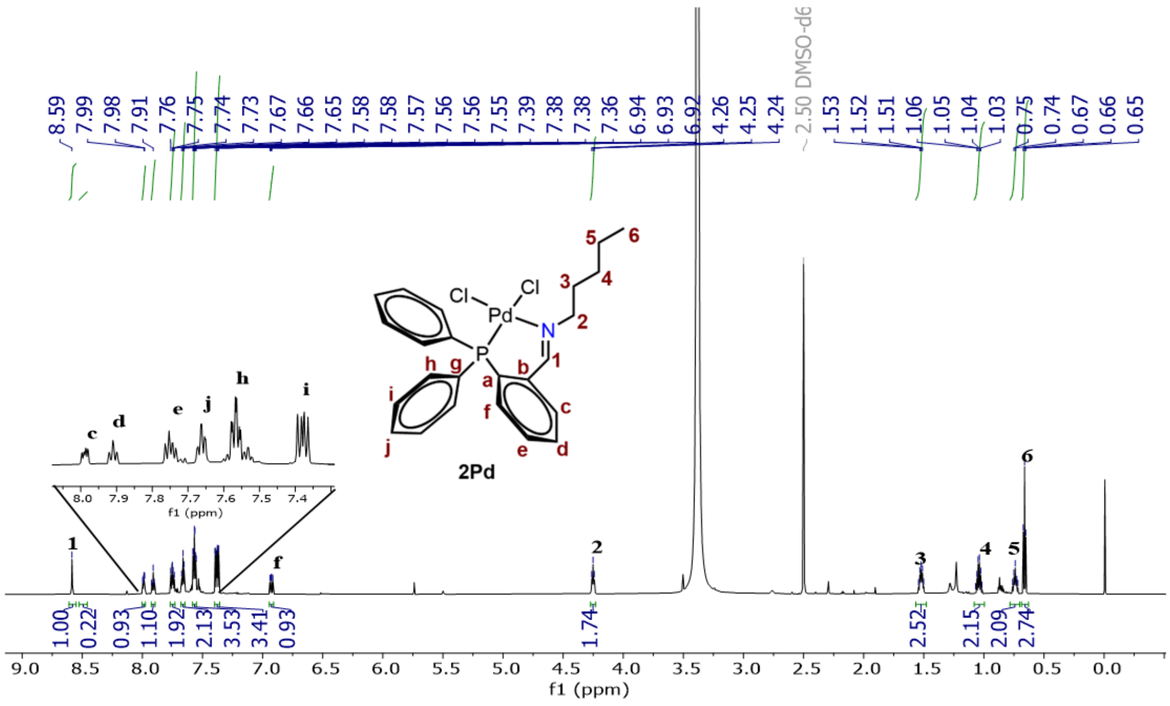

Figure S4. ^{1}H NMR of (**2Pd**) in DMSO-d6.

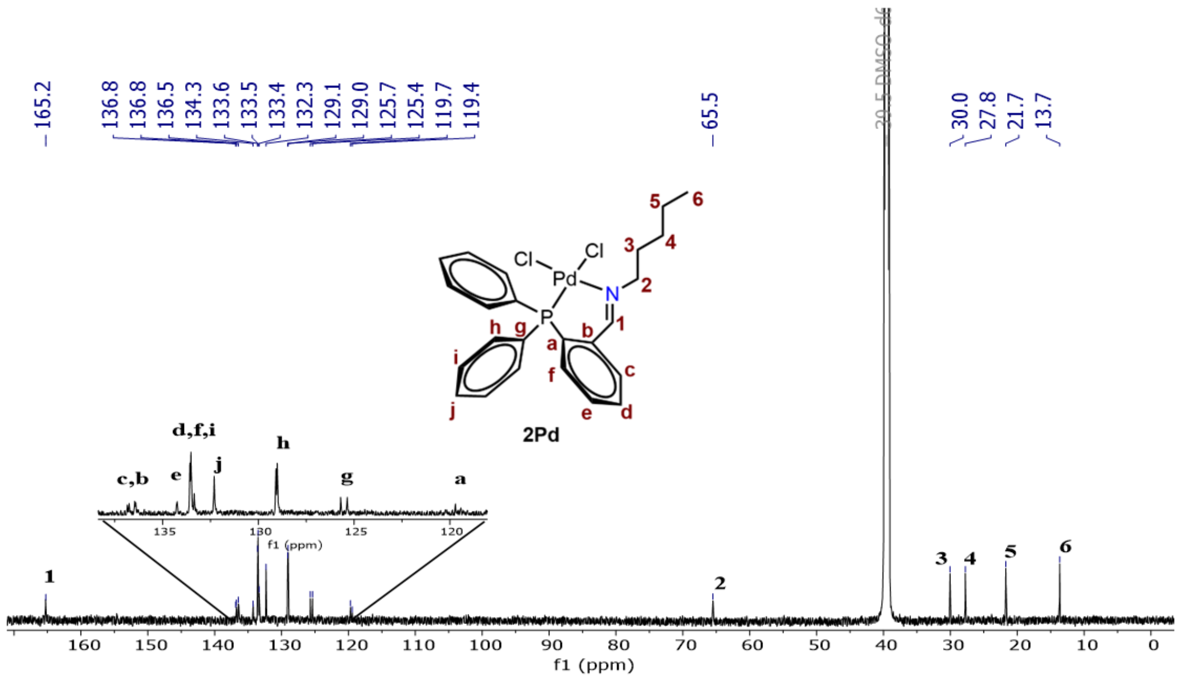

Figure S5. $^{13}C\{^{1}H\}$ NMR of (**2Pd**) in DMSO-d6.

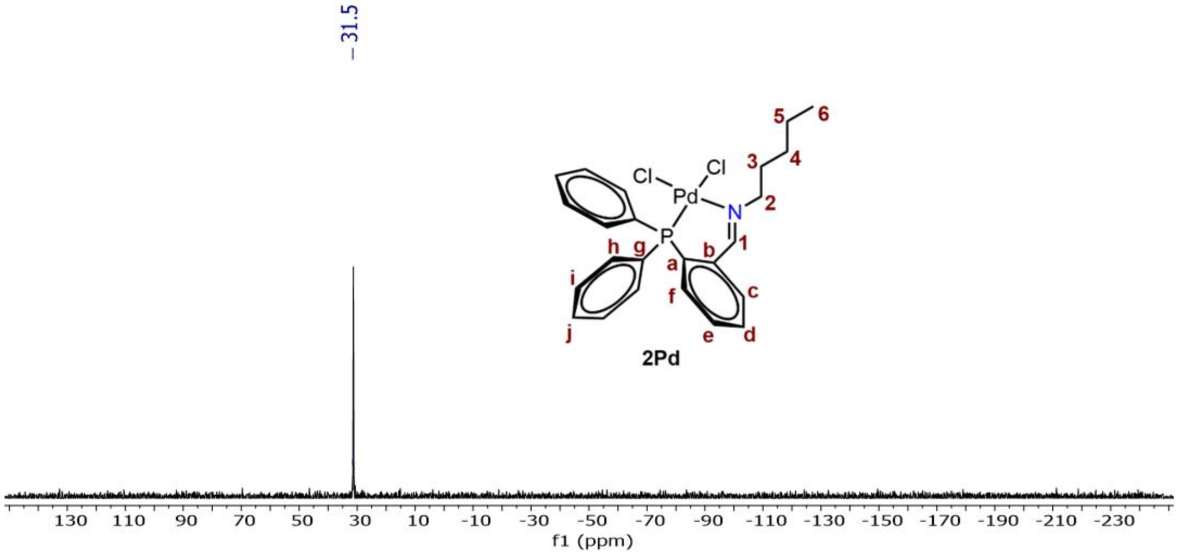

Figure S6. $^{31}P\{^{1}H\}$ NMR of (**2Pd**) in DMOS-d6.

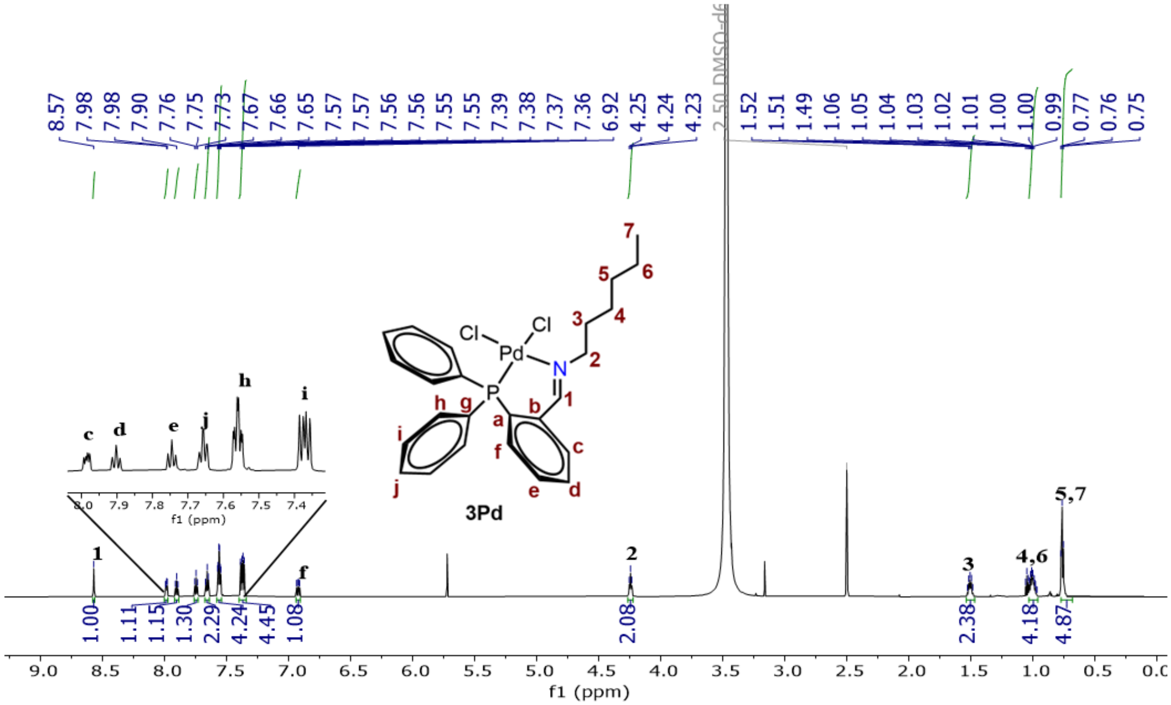

Figure S7. ^{1}H NMR of (**3Pd**) in DMSO-d6.

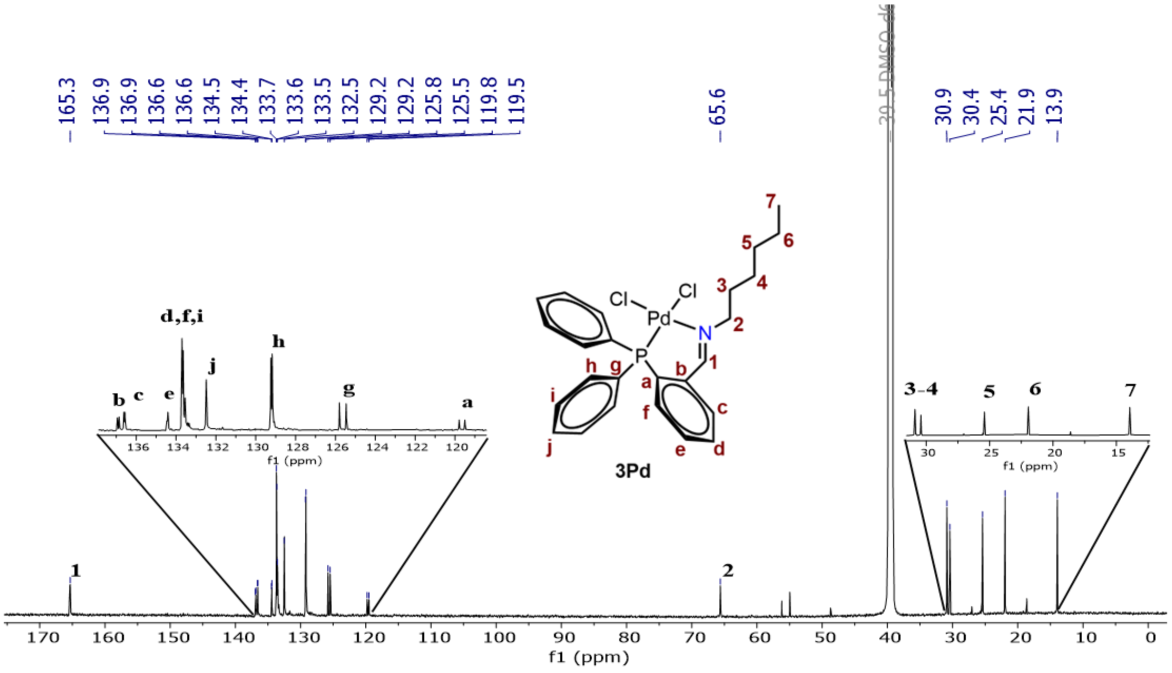

Figure S8. $^{13}C\{^1H\}$ NMR of (**3Pd**) in DMSO-d$_6$.

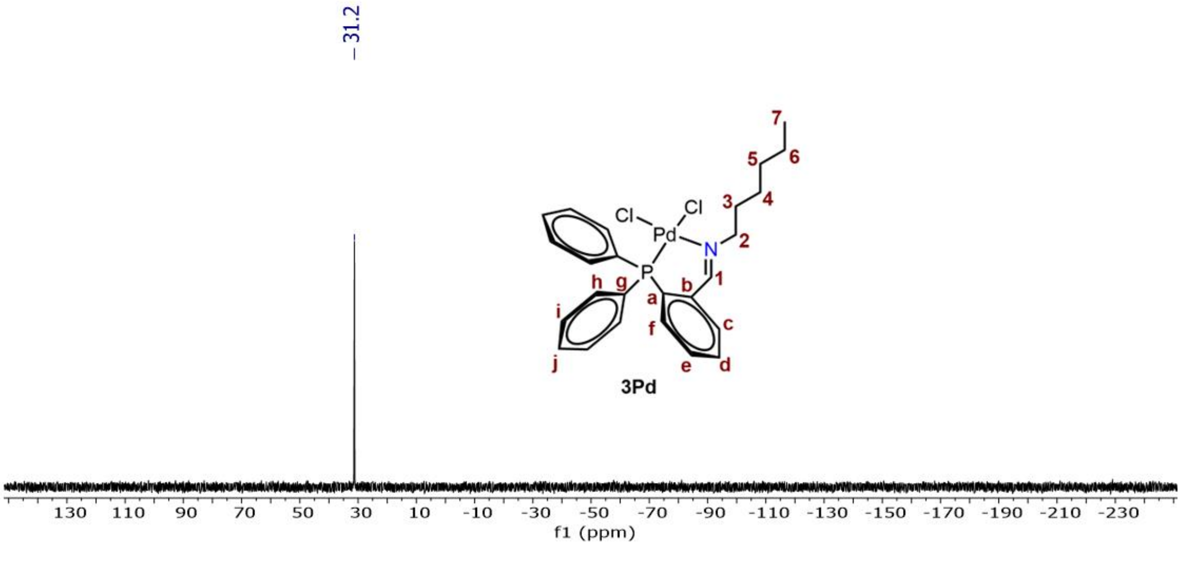

Figure S9. $^{31}P\{^1H\}$ NMR of (**3Pd**) in DMOS-d$_6$.

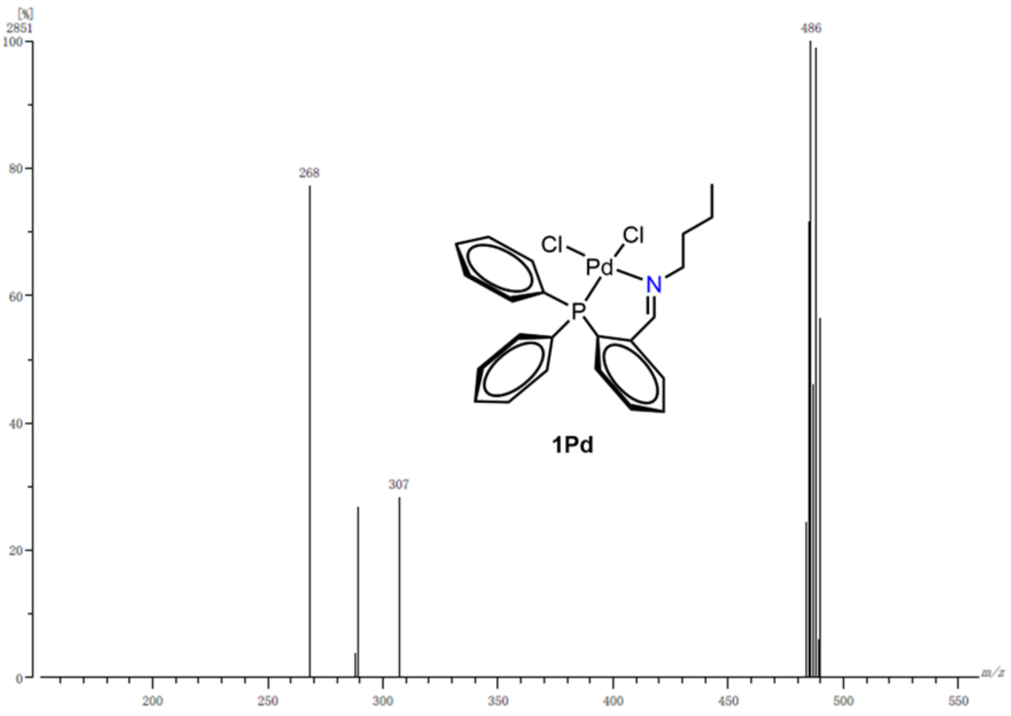

Figure S10. FAB$^+$-MS spectrum of (**1Pd**).

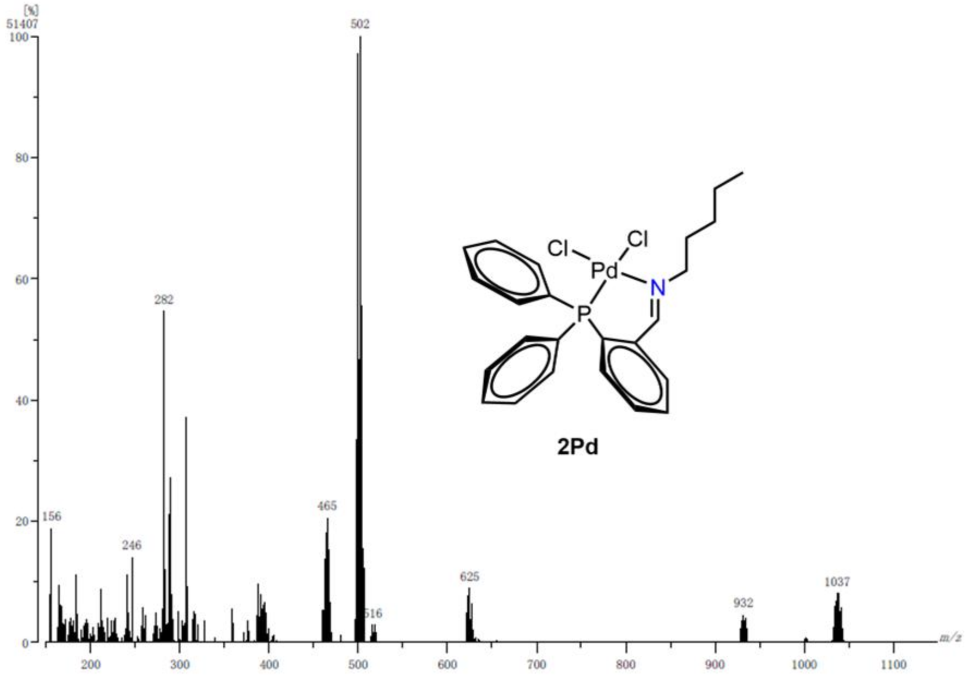

Figure S11. FAB$^+$-MS spectrum of (**2Pd**).

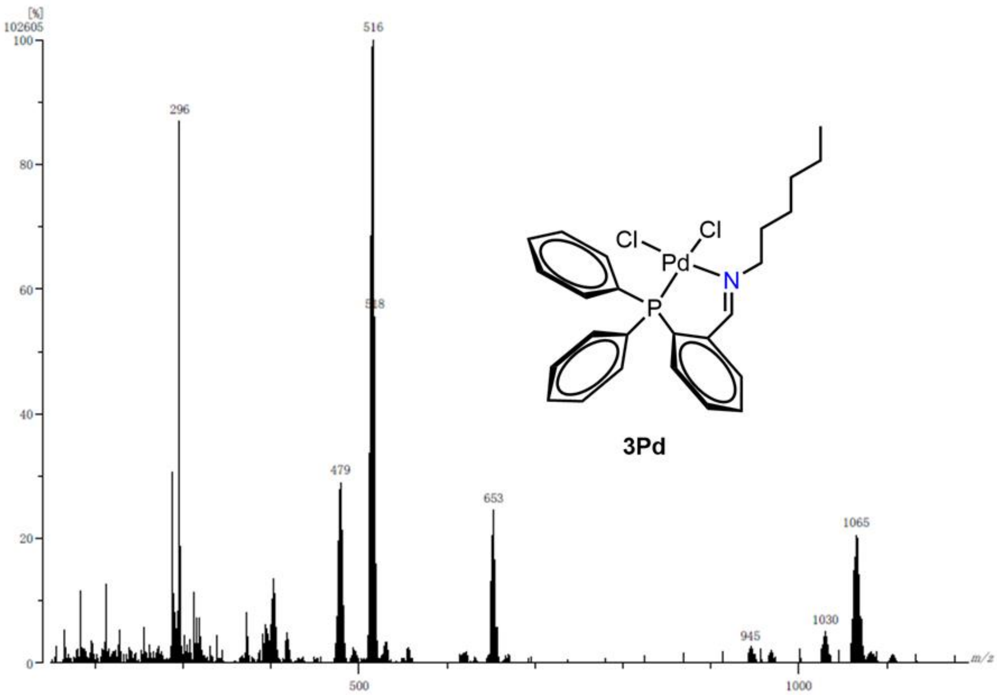

Figure S12. FAB$^+$-MS spectrum of (**3Pd**).

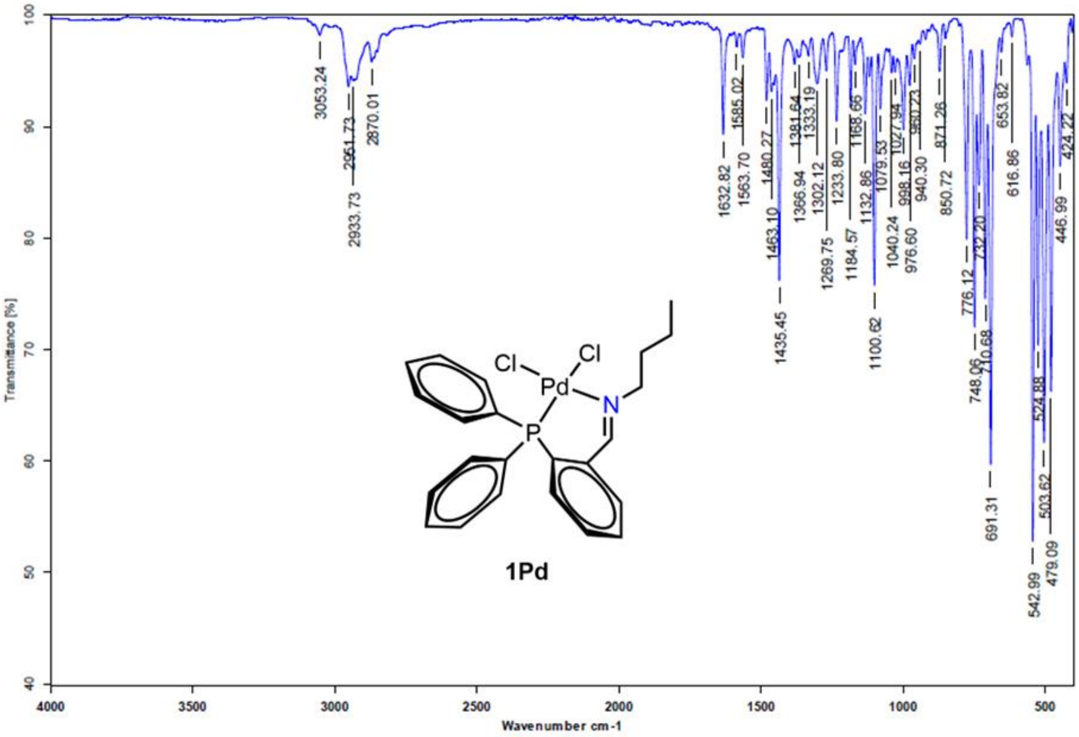

Figure S13. IR spectra of (**1Pd**).

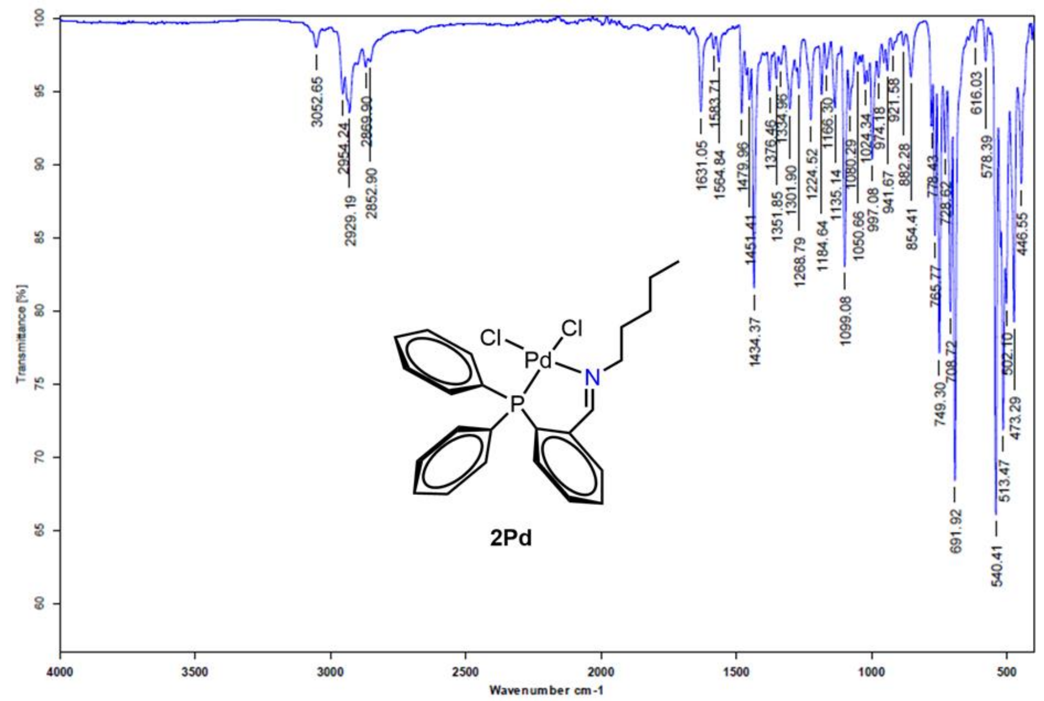

Figure S14. IR spectra of (**2Pd**).

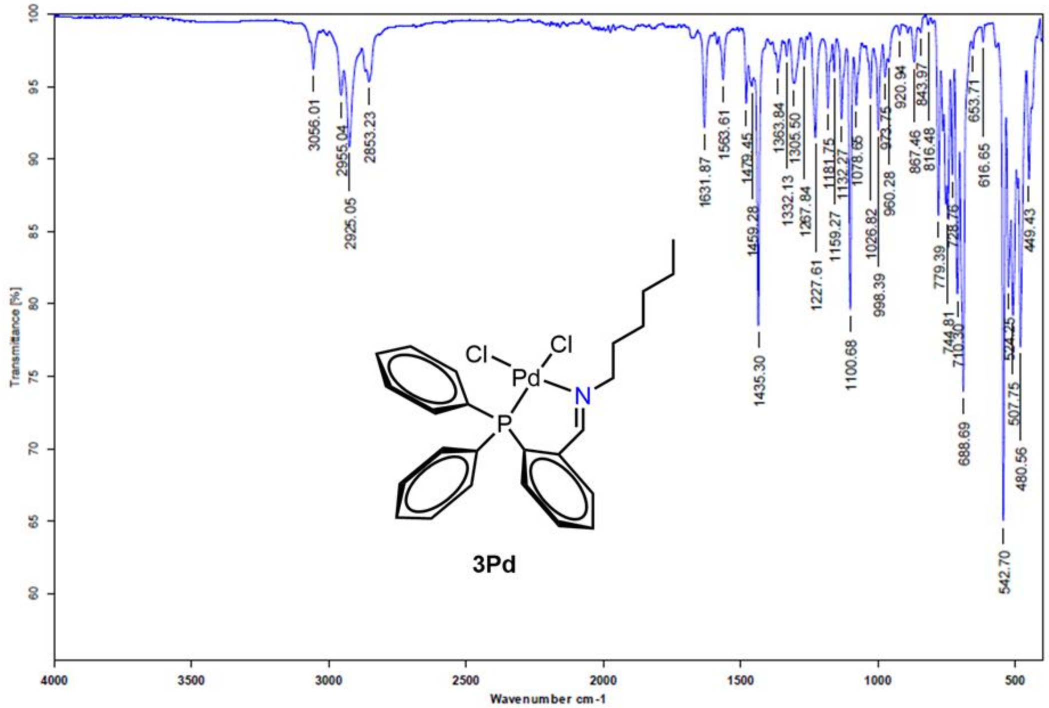

Figure S15. IR spectra of (**3Pd**).

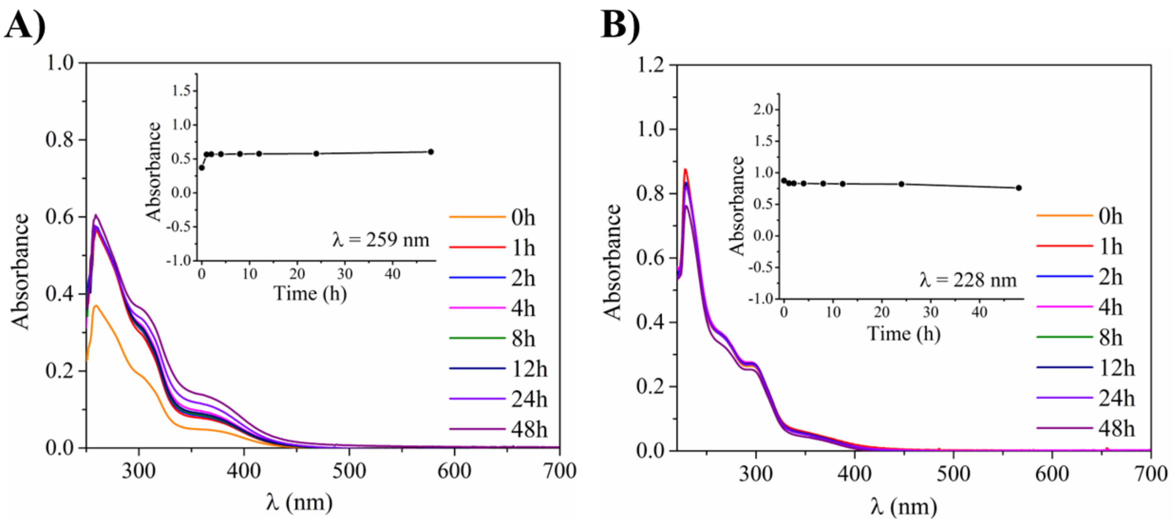

Figure S16. Stability assays by UV-Vis of the **1Pd A**) DMSO and **B**) with 0.1% (v/v) DMSO/H_2O).

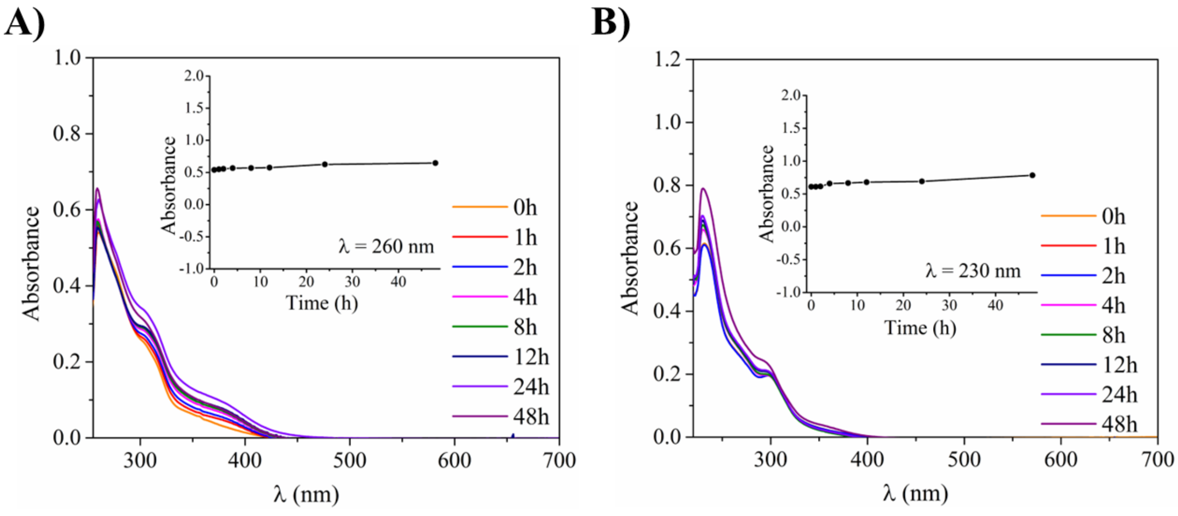

Figure S17. Stability assays by UV-Vis of the **2Pd A**) DMSO and **B**) with 0.1% (v/v) DMSO/H_2O).

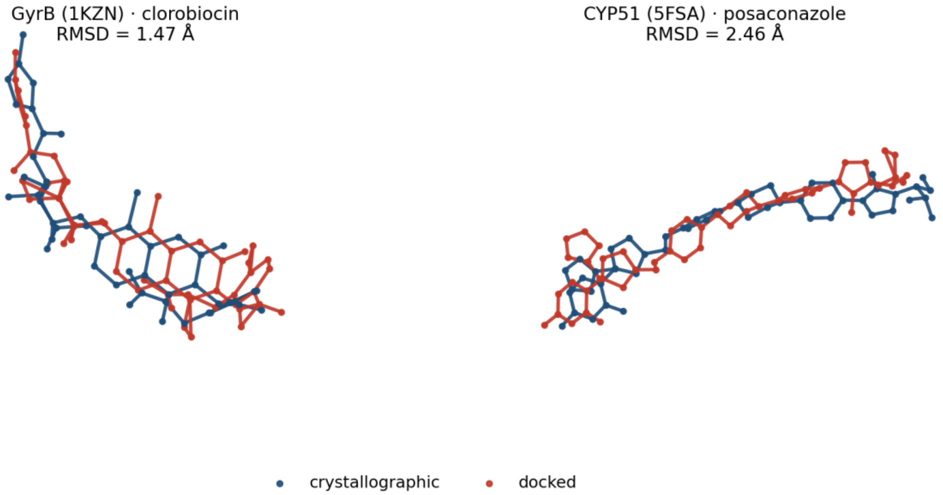

Figure S18. Superposition of the crystallographic conformation (blue) and the best docked pose (red) of the native ligand of each validated target. Coordinates are provided as 1KZN_superposition.pdb and 5FSA_superposition.pdb, in which chain A is the crystallographic conformation and chain B the docked pose.

Table S2. Cartesian coordinates, total energies and residual forces of the relaxed structures of complexes **1Pd-3Pd**.

Geometries relaxed with ORCA 6.0.1 at the PBE/def2-TZVP level (def2-ECP on Pd) with CPCM(water) implicit solvation, through the Atomic Simulation Environment interface implemented in MetalDock. All complexes are neutral, closed-shell species (charge = 0, S = 0). Relaxation was carried out until the maximum residual force fell below 0.05 eV Å−1. Vibrational frequencies were not computed, so the structures are reported as relaxed geometries rather than as vibrationally characterized minima. Coordinates in Å.

Complex	Atoms	Formula	E (Hartree)	max |F| (eV Å^{-1})
1Pd	52	C23H24Cl2NPPd	-2334.121521	0.0478
2Pd	55	C24H26Cl2NPPd	-2373.391983	0.0376
3Pd	58	C25H28Cl2NPPd	-2412.662130	0.0438

Complex 1Pd — 52 atoms, E = -2334.121521 Hartree

Pd 1.68344106 6.14886142 4.79932672

Cl 0.37182517 7.18211200 3.17789498

Cl 0.58763164 4.05294918 4.27467225

P	2.93506298	7.99240741	5.01311692
N	2.67864787	5.31746559	6.41056234
C	4.92133875	6.34754014	6.15637921
C	4.65502030	7.46579555	5.32938439
C	5.72640451	8.21162522	4.82221588
H	5.53354044	9.07436679	4.18438430
C	7.04767003	7.87192393	5.12922845
H	7.86509544	8.46630254	4.71802682
C	7.31143356	6.79706479	5.97810667
H	8.33720270	6.54124390	6.24324304
C	6.25438954	6.04988506	6.49224128
H	6.45101812	5.21152905	7.16281244
C	3.91027306	5.48687033	6.76112877
H	4.26605559	4.90696581	7.62396344
C	2.44797221	9.03348157	6.43080699
C	3.35459575	9.41180359	7.43262617
H	4.40301823	9.11628381	7.37251126
C	2.91131895	10.16293697	8.52496073
H	3.61807910	10.44091313	9.30874048
C	1.57229191	10.54937975	8.61601492
H	1.22797020	11.12649587	9.47585486
C	0.67057335	10.18805167	7.60934118
H	-0.37660688	10.48618045	7.67724166
C	1.10095443	9.42562706	6.52413347
H	0.38992744	9.12643004	5.75091361
C	3.06125861	9.10075819	3.56940866
C	3.36048493	8.54276020	2.31467009
H	3.45756323	7.46095607	2.20737451
C	3.52561911	9.36920131	1.20478677
H	3.75997187	8.92905142	0.23407449
C	3.38221005	10.75598877	1.33247624
H	3.50788322	11.40055618	0.46062042
C	3.07659781	11.31293009	2.57562699

H	2.96306535	12.39354946	2.68087047
C	2.91976144	10.48979972	3.69577738
H	2.69336310	10.93659777	4.66446639
C	1.85195420	4.44998617	7.27826952
H	2.51357469	3.97535521	8.02074581
H	1.41419653	3.67748290	6.63327771
C	0.72898153	5.21392274	7.98503413
H	0.11766179	5.73940551	7.23091561
H	0.07099767	4.45725206	8.44439103
C	1.19765394	6.19540343	9.05922509
H	1.88944374	6.93027738	8.61727465
H	1.77488435	5.64870173	9.82492836
C	0.02953317	6.93116100	9.71833264
H	-0.55033226	7.49424159	8.97057556
H	-0.65695369	6.22749808	10.21491624
H	0.37980533	7.64947270	10.47410778

Complex 2Pd — 55 atoms, E = -2373.391983 Hartree

Pd	1.69878679	6.13627928	4.76921903
Cl	0.40159206	7.14647934	3.11660995
Cl	0.63685001	4.02086727	4.25240486
P	2.93924773	7.99228473	5.01007882
N	2.67622546	5.31591373	6.38984029
C	4.92460704	6.32867326	6.13907098
C	4.65822614	7.46850727	5.34305316
C	5.73037340	8.23180816	4.86557669
H	5.53986388	9.12270657	4.26680196
C	7.05115683	7.87482592	5.15437470
H	7.86888186	8.48572444	4.76903120
C	7.31641685	6.75662463	5.94599167
H	8.34418855	6.47708665	6.18063535
C	6.25796814	6.00345771	6.44921030
H	6.45387660	5.14043227	7.08954765
C	3.90599625	5.48111605	6.74682896

H	4.25046493	4.91574021	7.62322346
C	2.44310694	9.04125790	6.41877322
C	3.36268907	9.46880407	7.38847783
H	4.41105967	9.17470485	7.32680495
C	2.93629552	10.27798786	8.44453212
H	3.65605264	10.60216731	9.19802252
C	1.59881880	10.67034211	8.53365843
H	1.26961338	11.30136203	9.36107069
C	0.68060937	10.25008287	7.56515377
H	-0.36456426	10.55703794	7.63140842
C	1.09577247	9.43077290	6.51491589
H	0.37475497	9.09477115	5.76638537
C	3.07591104	9.10209152	3.56563552
C	3.38027773	8.54434587	2.31200632
H	3.49047655	7.46362760	2.20713380
C	3.53185252	9.36912397	1.19866785
H	3.76754092	8.92845441	0.22843392
C	3.37156151	10.75434249	1.32222486
H	3.48113193	11.39651950	0.44631906
C	3.07035236	11.31188807	2.56608922
H	2.95121873	12.39186854	2.66927860
C	2.92308771	10.49023770	3.68864521
H	2.69449451	10.93782404	4.65618770
C	1.84346406	4.45144684	7.25452631
H	2.50106670	3.96002719	7.99030589
H	1.39981686	3.68772641	6.60209935
C	0.72643617	5.21480420	7.97467032
H	0.14065448	5.78477417	7.23204085
H	0.04670667	4.44886508	8.38512874
C	1.19250626	6.13503957	9.10572568
H	1.90777461	6.88056410	8.71853241
H	1.73806434	5.53744440	9.85848956
C	0.03115931	6.86640790	9.78607305

H	-0.49214002	7.48194559	9.03472453
H	-0.70481546	6.12335251	10.13773564
C	0.46621437	7.74962937	10.95567946
H	0.93414124	7.15244185	11.75332224
H	1.19479148	8.50882320	10.63160109
H	-0.39163836	8.27844415	11.39621267

Complex 3Pd — 58 atoms, E = -2412.662130 Hartree

Pd	1.69406238	6.13844979	4.76483026
Cl	0.39180769	7.15708049	3.12217308
Cl	0.63026515	4.02398382	4.23678004
P	2.93874246	7.99252190	5.00830059
N	2.67198233	5.30825716	6.38197428
C	4.92121040	6.32604671	6.13669613
C	4.65697906	7.46561349	5.33972892
C	5.72897919	8.22828175	4.86222264
H	5.53547462	9.11603833	4.25961524
C	7.04879608	7.87219501	5.15494456
H	7.86416363	8.48508848	4.77043907
C	7.31392745	6.75813001	5.95208494
H	8.34202002	6.48405232	6.19219618
C	6.25451988	6.00363067	6.45143223
H	6.45093918	5.14371688	7.09504087
C	3.90222868	5.47339612	6.73807728
H	4.24958982	4.90482204	7.61101572
C	2.44432004	9.04143807	6.41806345
C	3.36383693	9.46358583	7.39017318
H	4.41219809	9.16820444	7.32784582
C	2.93606013	10.26874255	8.44867629
H	3.65537898	10.58849518	9.20381821
C	1.59917788	10.66365258	8.53703073
H	1.26914923	11.28931342	9.36811270
C	0.68199593	10.24837968	7.56553020
H	-0.36254302	10.55651755	7.63035853

C	1.09738135	9.43288025	6.51254570
H	0.37799554	9.10064355	5.76041530
C	3.07385603	9.10214476	3.56338316
C	3.37741818	8.54405791	2.30961072
H	3.48601275	7.46297939	2.20496822
C	3.53060362	9.36911371	1.19659759
H	3.76805503	8.92845828	0.22678480
C	3.37322104	10.75477391	1.32142181
H	3.48376533	11.39759258	0.44612228
C	3.07112866	11.31197739	2.56553067
H	2.95400905	12.39186232	2.67054619
C	2.92311390	10.49035154	3.68809360
H	2.69523410	10.93631716	4.65693021
C	1.84102639	4.44113409	7.24618699
H	2.49925822	3.94337244	7.97611928
H	1.39242658	3.68087730	6.59488503
C	0.73199129	5.20306569	7.97726249
H	0.13434615	5.76885530	7.24215856
H	0.05990684	4.43786814	8.40019420
C	1.21555228	6.12880416	9.09456914
H	1.93722145	6.85995232	8.69372058
H	1.75980333	5.53402953	9.84972746
C	0.07208373	6.88835781	9.77302613
H	-0.44347018	7.51052941	9.02030331
H	-0.67871441	6.16765978	10.14173407
C	0.52595577	7.78001496	10.93151025
H	0.95423844	7.14950193	11.72928669
H	1.34489001	8.43240094	10.58348343
C	-0.59328060	8.64756537	11.51276699
H	-0.99985080	9.33030116	10.74899172
H	-0.22602807	9.26140313	12.35013767
H	-1.42641317	8.03051959	11.88682466

Table S3. CM5 atomic partial charges of the metal coordination sphere of complexes 1Pd–3Pd.

Charges derived from a Hirshfeld population analysis of the converged density and used directly in the AutoDock4 PDBQT files.

Complex	q(Pd)	q(Cl_1)	q(Cl_2)	q(P)	q(N)
1Pd	+0.7414	-0.5139	-0.5875	-0.0768	-0.3918
2Pd	+0.7397	-0.5182	-0.5845	-0.0740	-0.3925
3Pd	+0.7383	-0.5180	-0.5856	-0.0738	-0.3922

Table S4. Docking results and cluster analysis for complexes 1Pd–3Pd against the selected molecular targets.

Independent Lamarckian genetic-algorithm runs were performed for each complex–target pair (population 150, 2.5×10^6 energy evaluations, 27 000 generations, elitism 1, mutation rate 0.02, crossover rate 0.8) on a grid box of 80 points per dimension with 0.375 Å spacing (30 Å per side; 160 points and 60 Å for the B-DNA dodecamer). Poses were grouped by ranked cluster analysis with a 2.0 Å RMSD tolerance.

Target (PDB)	Complex	ΔG lowest	Mean ΔG top cluster	Runs in cluster	Clusters	Range
CYP51 (5FSA)	1Pd	-10.36	-10.17	41/50	7	4.56
CYP51 (5FSA)	2Pd	-10.63	-10.42	43/50	4	3.46
CYP51 (5FSA)	3Pd	-10.75	-10.52	33/50	10	4.85
GyrB (1KZN)	1Pd	-6.04	-5.74	4/50	11	1.20
GyrB (1KZN)	2Pd	-5.96	-5.88	8/50	12	1.16
GyrB (1KZN)	3Pd	-6.38	-5.94	9/50	13	1.74
PBP2a (1VQQ)	1Pd	-6.54	-6.31	49/50	2	1.00
PBP2a (1VQQ)	2Pd	-6.75	-6.27	47/50	3	2.77
PBP2a (1VQQ)	3Pd	-7.12	-6.60	46/50	4	2.14

Target (PDB)	Complex	ΔG lowest	Mean ΔG top cluster	Runs in cluster	Clusters	Range
B-DNA (1BNA)	1Pd	-5.65	-5.65	1/100	35	1.57
B-DNA (1BNA)	2Pd	-5.89	-5.85	2/100	49	2.00
B-DNA (1BNA)	3Pd	-5.66	-5.66	1/100	48	1.61

ΔG values in kcal mol^{-1}. "Range" is the spread of predicted binding free energies among the independent runs of the same complex–target pair.

Table S5. Redocking validation of the docking protocol.

The native ligand co-crystallized with each target was redocked into its own binding site using the same receptor model and the same grid box employed for complexes 1Pd–3Pd. RMSD values are symmetry-corrected and computed over heavy atoms against the crystallographic conformation.

Target (PDB)	Native ligand	Rotatable bonds	Sampling	Best RMSD (Å)
GyrB (1KZN)	clorobiocin (CBN)	13	2.5×10^7 evaluations, 10 runs	1.47
CYP51 (5FSA)	posaconazole (X2N)	13	2.5×10^6 evaluations, 50 runs	2.46

RMSD of the best pose among those generated for each target. For GyrB this pose was also the top-ranked solution. No co-crystallized ligand suitable for conventional redocking is available for PBP2a, whose deposited β-lactam complexes are covalent acyl-enzyme adducts, or for the B-DNA dodecamer.
